# Subtype-specific secretomic characterization of pulmonary neuroendocrine tumor cells

**DOI:** 10.1038/s41467-019-11153-5

**Published:** 2019-07-19

**Authors:** Xu-Dong Wang, Rongkuan Hu, Qing Ding, Trisha K. Savage, Kenneth E. Huffman, Noelle Williams, Melanie H. Cobb, John D. Minna, Jane E. Johnson, Yonghao Yu

**Affiliations:** 1 Department of Biochemistry, University of Texas Southwestern Medical Center, Dallas, 75390 TX USA; 2 Department of Neuroscience, University of Texas Southwestern Medical Center, Dallas, 75390 TX USA; 3 Simmons Comprehensive Cancer Center, University of Texas Southwestern Medical Center, Dallas, 75390 TX USA; 4 Department of Pharmacology, University of Texas Southwestern Medical Center, Dallas, 75390 TX USA; 5 Hamon Center for Therapeutic Oncology Research, University of Texas Southwestern Medical Center, Dallas, 75390 TX USA; 6 Department of Internal Medicine, University of Texas Southwestern Medical Center, Dallas, 75390 TX USA

**Keywords:** Proteomics, Lung cancer, Tumour biomarkers, Mass spectrometry

## Abstract

Pulmonary neuroendocrine (NE) cancer, including small cell lung cancer (SCLC), is a particularly aggressive malignancy. The lineage-specific transcription factors Achaete-scute homolog 1 (ASCL1), NEUROD1 and POU2F3 have been reported to identify the different subtypes of pulmonary NE cancers. Using a large-scale mass spectrometric approach, here we perform quantitative secretome analysis in 13 cell lines that signify the different NE lung cancer subtypes. We quantify 1,626 proteins and identify IGFBP5 as a secreted marker for ASCL1^High^ SCLC. ASCL1 binds to the E-box elements in *IGFBP5* and directly regulates its transcription. Knockdown of ASCL1 decreases IGFBP5 expression, which, in turn, leads to hyperactivation of IGF-1R signaling. Pharmacological co-targeting of ASCL1 and IGF-1R results in markedly synergistic effects in ASCL1^High^ SCLC in vitro and in mouse models. We expect that this secretome resource will provide the foundation for future mechanistic and biomarker discovery studies, helping to delineate the molecular underpinnings of pulmonary NE tumors.

## Abstract (web summary)

Secreted proteins present a rich resource of potential cancer biomarkers. Here, the authors use mass spectrometry to analyze secretome remodeling in pulmonary neuroendocrine lung cancer cell lines and validate potential biomarkers and therapeutic targets in vitro and in mouse models.

## Introduction

Lung cancer with neuroendocrine features (NE-lung cancer) is an aggressive type of lung carcinoma that accounts for about 25% of all lung cancer cases, including all SCLC, a subset of NSCLC, and typical and atypical carcinoids^1,2^. The biology of SCLC is distinct from the more common classic non-small cell lung cancer (NSCLC, including squamous cell carcinoma and adenocarcinoma), in that it originates from NE cells that often secrete low molecular weight polypeptide hormones and biogenic amines^3^. Even though representing a minor fraction of all lung cancer cases, SCLC is one of the most lethal human malignancies. At diagnosis, patients usually present with highly advanced, metastatic diseases. Accordingly, only 7% of patients are alive 5 years following the diagnosis of SCLC^1^. Based on these characteristics, SCLC has been identified as one of the two cancer types (the other is pancreatic ductal adenocarcinoma) to be included in the Recalcitrant Cancer Research Act (RCRA)^4^. NE-NSCLC is a subset of NSCLC tumors (∼10% of NSCLSs), which has NE features and a gene expression signature similar to that of SCLC^2,5^. Like the SCLC, NE-NSCLC is also a highly aggressive disease with very poor prognosis. These two types of NE-lung cancer had an unexplained but substantial increase in the United States in the last 30 years^6,7^. Approximately 30,000 SCLC cases are diagnosed annually in the US, which in fact makes it more common than many other cancers (e.g., myeloma, brain and esophagus)^8^.

Clinically, SCLC was previously regarded as a “homogenous” disease, which led to most SCLC patients being treated similarly (usually with platinum/etoposide doublet chemotherapy) in the past three decades^1^. However, recent studies have indicated that there is considerable heterogeneity among SCLCs in terms of expression of lineage-specific oncogenes (e.g., ASCL1, NEUROD1 and POU2F3)^5,9–11^, histology^12^, growth characteristics^13^, expression of NE cell differentiation markers^5,10^, MYC family member activation^14^, and mechanisms of Notch pathway inactivation^15,16^. These findings point to the critical need for better classification of the different SCLC subtypes, and accordingly more personalized treatment regimens.

Despite the recent progresses in our understanding of the molecular underpinnings of SCLC, treatment options remain limited, in part due to the lack of methods for early detection of this disease. Even though the pathogenesis of SCLC is driven by the above-mentioned lineage-specific oncogenes, these genes themselves are poor candidates as diagnostic biomarkers. Secreted proteins are known to provide an essential communication network between the cancer cells and their adjacent microenvironment. This has been shown to be an essential mechanism that promotes tumor growth, invasion, or angiogenesis^17^. Importantly, secreted proteins originating from the NE tumor can also enter systemic circulation^18^, and therefore, represent a rich resource for potential diagnostic and prognostic markers^19^.

Proteomic approaches are, arguably, the methods of choice to study the tumor secretome^20^. Recent advances in cancer cell secretome analysis showed the application of the proteomic approaches for the identification of secreted proteins^21,22^. Recently, using an improved approach based on multi-dimensional HPLC separation combined with high sensitivity mass spectrometry, we identified many low abundance secreted proteins from NSCLC cells^23^. Importantly, many of these proteins are involved in regulating biological processes in the extracellular space (e.g., those related to metastasis).

To better understand how the secretome is remodeled during the pathogenesis of NE-lung cancers, and to find potential biomarkers of NE-lung cancer, we perform quantitative mass spectrometric analysis to investigate the secretome of high-grade NE-lung cancers. Using several well-established cell line models, we report the secretome of the two major NE-lung cancer subtypes, i.e., ASCL1^High^ and NEUROD1^High^. We further show, both at RNA and protein levels, that the secreted protein IGFBP5 correlates with NE-lung cancer cells expressing ASCL1. Moreover, IGFBP5 is identified to be a direct transcriptional target of ASCL1. Because IGFBP5 is a potent inhibitor of IGF-1 signaling, therapeutically targeting ASCL1 leads to the downregulation of IGFBP5, which, in turn, causes the hyperactivation of the IGF-1 signaling pathway. Co-targeting of ASCL1 and IGF-1R signaling leads to marked synergistic growth inhibitory effects in ASCL1^High^ SCLC in vitro. We further validate the efficacy of the combination treatment in suppressing tumor growth in vivo using ASCL1^High^ SCLC xenograft and PDX models. Taken together, these observations show that the quantitative analysis of the NE-lung cancer secretome will provide a roadmap to identify potential biomarkers and therapeutic targets in high-grade NE-lung cancers.

## Results

### Comprehensive analysis of the NE-lung cancer secretome

**Fig. 1:**
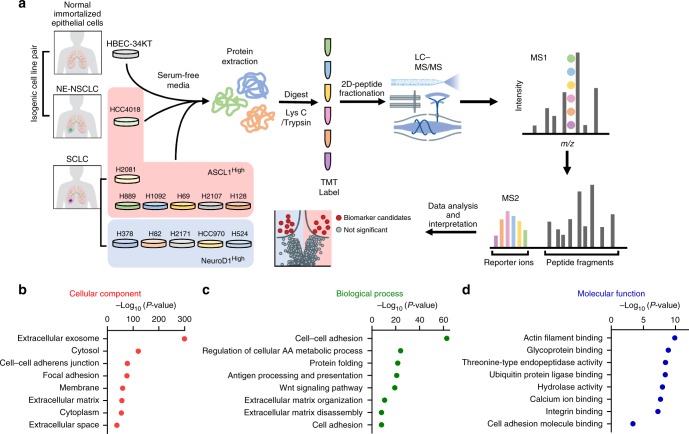
Quantitative mass spectrometric analysis of the neuroendocrine (NE)-lung cancer secretome. **a** Schematic overview of the quantitative proteomic platform. Briefly, proteins from the conditioned media (CM) were extracted and then digested with trypsin. The samples were labeled with the tandem mass tag (TMT) reagents. **b**–**d** Gene Ontology (GO) analysis of the proteins identified from the CM samples of the thirteen lung cell lines shown in **a**. Cellular component (**b**), biological processes (**c**), and molecular function (**d**) analyses were shown

To comprehensively analyze the secretome of high-grade NE-lung cancer, we sought to profile the secreted proteins in the conditioned media (CM) from a panel of 13 human lung cancer cell lines (Fig. 1a). This panel was composed of the following cell lines that signify the normal/different subtypes of NE-lung cancers: (1) a human bronchial epithelial cell line HBEC34-KT (immobilized by overexpressing TERT and CDK4) that is characterized by a number of properties consistent with untransformed epithelial cells, including: normal epithelial morphology, expression of epithelial markers, lacking anchorage-independent growth and inability to form tumors in vivo^24^; (2) HCC4018 (an ASCL1^High^ NE-NSCLC, which is derived from the same patient as of HBEC34-KT^25,26^); (3) additional ASCL1^High^ SCLC lines (*n* = 6, H2081, H889, H1092, H69, H2107, H128); (4) NEUROD1^High^ SCLC lines (*n* = 5, H378, H82, H2171, HCC970, H524). Thus, our secretomic analysis spanned a normal control and two high-grade NE-lung cancer subtypes, allowing for comprehensive understanding of secreted proteins involved in NE-lung cancers. These cell lines also allow the comparison of the secretome between cells with the same genomic background (HBEC34-KT and HCC4018)^26^, as well as cross-cancer subtypes of SCLC (ASCL1^High^ and NEUROD1^High^).

To determine the secreted proteins in these 13 cell lines, cells were starved in serum-free media (SFM) for 24 h. Then the CM was harvested and subjected to isobaric-labeling-based tandem mass tag (TMT) MS analysis. A schematic of the quantitative secretomic platform is shown in Fig. 1a. One major challenge associated with secretomic analysis is the low abundance of many secreted signaling proteins, and hence the potentially large dynamic range for their detection^19^. To facilitate deep sequencing of these secreted proteins, the pooled peptides were subjected to off-line two-dimensional HPLC separation and were analyzed by high sensitivity MS and MS/MS experiments^23^. To allow the cross-reference analysis of multiple datasets, these TMT experiments were performed under identical cellular conditions. Three sets of TMT experiments were performed to characterize the aforementioned 13 cell lines, with HBEC34-KT cells included in all three TMT sets as a reference standard. From these three TMT sets, we were able to identify and quantify a total of 6616 proteins (false discovery rate = 0.45%), among which 1626 proteins were commonly identified and quantified across the 13 cell lines (Supplementary Data 1–3). The biological replicate samples in one TMT set (HBEC34-KT, HCC4018, and H2081, Supplementary Data 1) showed high correlation in protein expression levels (Supplementary Fig. 2b–d), validating the reproducibility of our quantitative mass spectrometric experiments.

To more comprehensively evaluate the dataset, we analyzed these 1626 commonly identified proteins in Gene Ontology (GO) database. GO cellular component analysis showed that 66.8% of proteins were mapped to the extracellular or membrane region, including compartments such as extracellular region (*P* *=* 4.72 × 10^−300^, Fisher’s exact test), cell–cell adherens junction (*P* *=* 2.04 × 10^−78^), membrane (*P* *=* 4.07 × 10^−60^), and extracellular matrix (*P* *=* 3.58 × 10^−57^) (Fig. 1b, Supplementary Fig. 1d, e), confirming the specificity of secretomic analyses. In line with this, further GO analysis indicated that these proteins were involved in biological processes related to the extracellular matrix biology, including cell adhesion (*P* *=* 1.8 × 10^−63^), antigen processing and presentation (*P* *=* 1.7 × 10^−21^), extracellular structure organization (*P* *=* 2.2 × 10^−11^), and molecular functions like actin filament binding (*P* *=* 1.3 × 10^−10^) (Fig. 1c, d). These results validated our method of secretome-focused proteomics and suggested that our dataset is enriched with secreted proteins with physiological functions. Of note, we detected a negligible amount of  lactate dehydrogenase (LDH) activity in the CM of all the cell lines used in the secretome analysis (Supplementary Fig. 1f), indicating that the proteins that are non-specifically released from cells as a result of apoptosis constitute a minor part of the overall proteins identified in our secretome analysis.

### Comparison of the secretome between NE-lung cancer and NSCLC

Previously, we performed quantitative proteomic analysis of the secretome of classic NSCLC cells^23^. Specifically, we identified and quantified 2713 secreted proteins from two isogenic NSCLC cell lines (NCI-H1993 and NCI-H2073), as well as an immortalized human bronchial epithelial cell line (HBEC3-KT). To get a deeper understanding of the current NE-lung cancer secretome dataset and its difference from the classic NSCLC secretome, we performed cross-reference analysis of the HBEC34-KT-HCC4018-H2081 secretome dataset with our previous NSCLC secretome dataset. The results indicated that there was a substantial overlap in the two datasets, with 1491 proteins commonly identified by both studies (Supplementary Fig. 2a, Supplementary data 4). At the same time, there were also 552 proteins that appeared to be selectively secreted by NE-lung cancer cells (Supplementary Fig. 2a). Further GO biological process analysis showed that these 552 proteins were enriched in the biological processes that are linked to neuron development, including neuron projection development (*P* *=* 6.7 × 10^−6^, Fisher’s exact test), axonogenesis (*P* *=* 1.5 × 10^−5^), neuron differentiation (*P* *=* 2.3 × 10^−4^), neuron projection morphogenesis (*P* *=* 5.7 × 10^−5^), and synapse organization (*P* *=* 6.6 × 10^−5^) (Supplementary Fig. 2b).

We also analyzed the expression of the 1491 proteins that are commonly present in the CM from both NSCLC and NE- lung cancer cells. Pairwise Pearson’s correlation analysis of the protein expression was performed for the six cell lines (i.e., HBEC3-KT, H2073, H1993, HBEC34-KT, HCC4018, and H2081). The results showed that the two normal cells (HBEC3-KT and HBEC34-KT) from two independent datasets displayed a highly similar secretome profile with a Pearson’s correlation coefficient of 0.99 (Supplementary Table 1). Using the two normal HBEC cell lines as the internal standard, we performed cross-reference analysis of the secretome profiles of the classic NSCLC and NE-lung cancer cells. Intriguingly, the two isogenic NSCLC cells (H2073 and H1993) formed a cluster, and so did the two NE-lung cancer cells (HCC4018 and H2081) (Supplementary Fig. 2b). However, these three clusters were distinct from each other. GO biological process analysis indicated that proteins over-secreted in H2073 and H1993 CM were enriched in regulation of cell motion (*P* *=* 5.4 × 10^−5^), regulation of cell migration (*P* *=* 1.1 × 10^−3^), and response to wounding (*P* *=* 3.4 × 10^−3^). On the other hand, proteins over-secreted in HCC4018 were linked to endocytosis (*P* *=* 0.014) and membrane invagination (*P* *=* 0.014) (Supplementary Fig. 2d). These data revealed the differences in protein secretion between classic NSCLC and NE-lung cancer, providing a potential link between the specific secretome of NE-lung cancer and its distinct biology compared to classic NSCLC.

### Generation of a NE-lung cancer-specific secretome signature

**Fig. 2:**
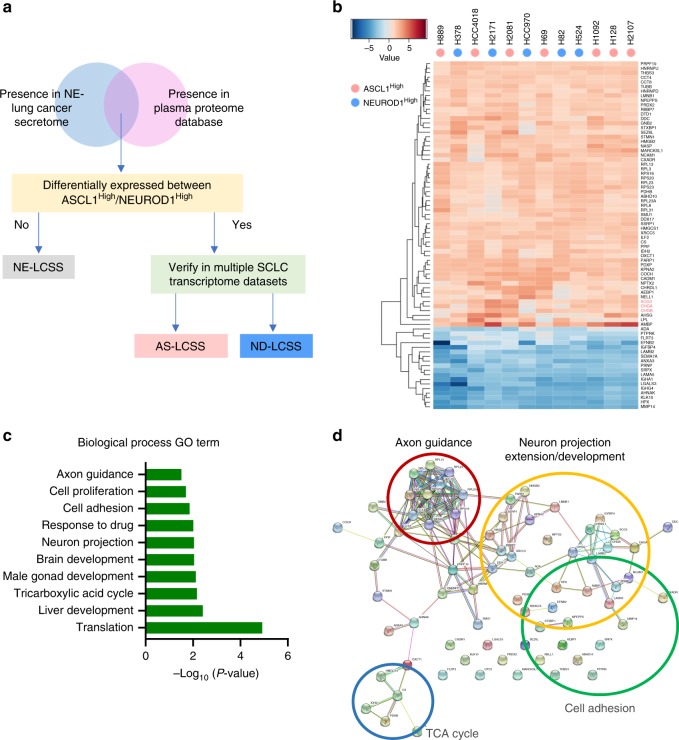
Generation of a NE-lung cancer-specific secretome signature (NE-LCSS). **a** Overview of the methodology to prioritize the candidate biomarkers for NE-LCSS, AS/ND-LCSS. **b** Heatmap showing the expression of the NE-LCSS across the 12 NE-lung cancer cell lines used in the secretome MS analysis. Pink dots indicate the ASCL1^High^ cells, and blue dots indicate the NEUROD1^High^ lines. Three known secreted markers for NE-Lung cancer (SCG3, CHGA, and CHGB) are highlighted in red. **c** *P-*value of the highly represented GO biological process terms for the NE-LCSS. **d** Protein–protein interaction network of NE-LCSS. Source data are provided as a Source Data file

In addition to defining the biological differences between NSCLC and NE-lung cancer at the secretome level, our TMT approach provides insights into the potential secreted biomarkers for high-grade NE-lung cancer in general, and furthermore, for the ASCL1^High^ and NEUROD1^High^ subtypes. To prioritize our candidates, a combination of statistical and biological filtering was used, including: (1) presence in the human plasma-proteome database (HUPO)^27^, (2) differential expression between these two subtypes of NE-lung cancer, and (3) unsupervised clustering analysis using previously published transcriptome datasets (Fig. 2a).

Although the majority of the identified proteins in the NE-lung cancer secretome were mapped to the extracellular region or membrane in GO analysis, the secretome dataset was further refined by cross-examination against the publicly available HUPO^27^ to identify proteins that have been detected in human plasma samples. To achieve this, we queried the 1626 proteins in the NE-lung cancer secretome dataset against HUPO (https://www.hupo.org/plasma-proteome-project), which yielded 1232 secreted proteins that could serve as potential biomarkers.

As HBEC34-KT cells were included in all TMT sets as a reference standard, the secretomic protein expression profile of each NE-lung cancer cell line was normalized to that of the HBEC34-KT cells (fold change, FC), in order to identify the proteins that are commonly upregulated or downregulated in the secretome of NE-lung cancer. We generated the upregulated NE-lung cancer-specific secretome signatures (NE-LCSS) by considering the proteins that: (1) are upregulated (average fold change for all NE-lung cancer cell lines) by at least 1.5-fold compared to the control HBEC34-KT cell line, and (2) are not differentially present in the secretome between the ASCL1^High^ and NEUROD1^High^ subtypes (log2 AveFC_ASCL1_/AveFC_NEUROD1_ < 0.2). The downregulated NE-LCSS was generated similarly. This analysis yielded a list of 76 proteins, including 58 upregulated and 18 downregulated proteins that shared the similar expression pattern in the secretome across the 13 NE-lung cancer lines compared to the normal control HBEC34-KT cells (Fig. 2b). Of note, some of these proteins have been reported as biomarkers for tumors with NE features, including CHGA, CHGB, and SCG3^28^. The identification of these known NE markers in the upregulated NE-LCSS demonstrated the validity of our approach.

We then analyzed the interaction networks of NE-LCSS to understand how these diverse sets of proteins are functionally interconnected. By using the STRING database^29^, we found these proteins formed well-linked networks that clustered into connected nodes based on protein families or biological processes. Specifically, we found several enriched biological processes that are related to neuron development, including brain development, neuron projection development, and axon guidance (Fig. 2c, d), suggesting a potential functional link between these aberrantly secreted proteins and the pathogenesis of NE-lung cancer.

### Generation of a subtype-specific LCSS for NE-lung cancer

The basic helix–loop–helix (bHLH) transcription factors ASCL1 and NEUROD1 are considered as the master regulator for a large fraction of the NE-lung cancers^30^. Based on the expression of these two transcription factors, NE-lung cancers can be divided into the ASCL1^High^, NEUROD1^High^, and double-negative subtypes. ASCL1^High^ NE-lung cancers share many similarities with NEUROD1^High^ SCLC in terms of histologic structures and immunohistochemical staining characteristics. However, it has also been shown that ASCL1 and NEUROD1 function as independent lineage-specific oncogenes that drive distinct gene expression programs, and thereby influence the pathogenesis, prognosis, and therapeutic responses of each individual SCLC case. The transcriptome of NE-lung cancers that is regulated by ASCL1 or NEUROD1 has been well studied^5,10,16,31^. However, the secretome program that signifies the ASCL1^High^ and NEUROD1^High^ NE-lung cancer subtypes has so far remained poorly defined.

**Fig. 3:**
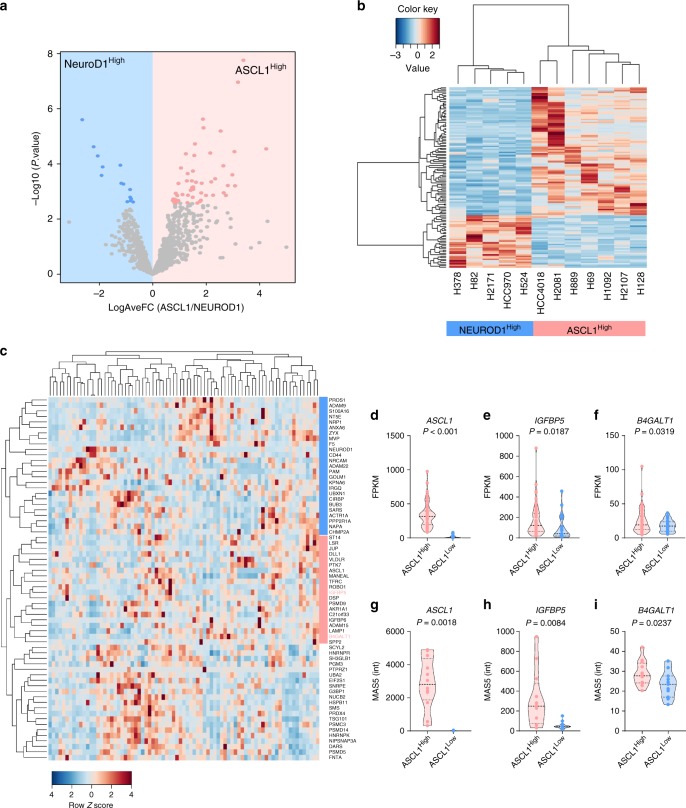
Generation of an LCSS for the ASCL1^High^ and NEUROD1^High^ NE-Lung cancer subtypes. **a** The volcano plot showing the indicated fold changes and *P*-values derived from t-test statistic. Proteins differentially present in the secretome between ASCL1^High^ and NEUROD1^High^ cells with an adjust *P*-value (FDR) < 0.05 are highlighted in pink and light blue, respectively. **b** The relative fold changes of the 65 significantly regulated secreted proteins in the secretome of 12 cell lines were grouped by unsupervised hierarchical clustering. **c** Transcriptomic clustering of 69 human SCLC tumors^16^ based on the AS/ND-LCSS gene expression markers (the pink bar indicates the AS-LCSS genes that are clustered with ASCL1, whereas the blue bar indicates the ND-LCSS genes that are clustered with NEUROD1). **d**–**f** Expression levels (FPKM) of ASCL1 (**d**), IGFBP5 (**e**), and B4GALT1 (**f**) in ASCL1^High^ (*n* = 54) and ASCL1^Low^ (*n* = 25) SCLC patient samples^16^. **g**–**i** Expression of ASCL1 (**g**), IGFBP5 (**h**), and B4GALT1 (**i**) in ASCL1^High^ (*n* = 12) and ASCL1^Low^ (*n* = 11) SCLC patient samples^33^. MAS5 intensity value averages of all probes for each gene in ASCL1^High^ and ASCL1^Low^ samples are indicated. Unpaired, two-tailed, Welch correction *t*-test results are indicated alongside the violin plots. The horizontal lines indicate the median and quartiles. Source data are provided as a Source Data file

With the fold change value (FC) we obtained for the secretome of each cell line, we used Limma^32^ to perform a direct comparison of the proteins present in the secretome of the ASCL1^High^ NE-lung cancer cell lines (*n* = 7) versus the NEUROD1^High^ NE-lung cancer cell lines (*n* = 5). This analysis identified a total of 65 proteins that were differentially present in the secretome between the two NE-Lung cancer subtypes (*P*_adj_ < 0.05, Benjamini–Hochberg correction) (Fig. 3a). We then performed unsupervised hierarchical clustering and the results showed that these 65 proteins were able to clearly separate the ASCL1^High^ group from the NEUROD1^High^ group (Fig. 3b), forming the ASCL1/NEUROD1 lung cancer secretome signature (AS/ND-LCSS).

Next, we asked whether the observed AS/ND-LCSS was driven by these lineage-specific transcription factors in the ASCL1^High^ and NEUROD1^High^ cancer subtypes. We extracted the mRNA expression data of AS/ND-LCSS genes and also *ASCL1* and *NEUROD1* from the previously published genome-wide microarray dataset in 39 NE-lung cancer cell lines^5^ (60 AS/ND-LCSS genes were found in these microarray data). The panel of 39 cell lines included 27 ASCL1^High^ and 12 NEUROD1^High^ lines. We used unsupervised hierarchical clustering to capture the unique feature of the expression of these 60 genes in these cell lines (Supplementary Fig. 3a). Specifically, clustering cell lines based on their AS/ND-LCSS expression profiles revealed the similarity among the ASCL1^High^ cells (i.e., HCC4018 and the 26 ASCL1^High^ SCLC lines), suggesting ASCL1^High^ NE-NSCLC and SCLC shared a more similar secreted gene expression phenotype. The 12 NEUROD1^High^ cell lines were also grouped together based on the expression of these 60 AS/ND-LCSS genes (Supplementary Fig. 3a). These data suggest that AS/ND-LCSS are able to separate the ASCL1^High^ cell lines from the NEUROD1^High^ lines in a larger panel of human lung cancer cell lines.

To further validate the relevance of the discovered AS/ND-LCSS, we analyzed two published transcriptome datasets obtained from human SCLC samples^16,33^. Consistent with our results obtained in cell lines, clustering analysis using the same AS/ND-LCSS further supported the separation of the human SCLC cohort into two subtypes, although a more moderate degree of separation was observed, likely due to the heterogeneity of SCLC patient samples (Fig. 3, Supplementary Fig. 3b). After reviewing these data, two genes (*IGFBP5*, *B4GALT1*) and one gene (*ANXA6*) were found to be consistently clustered with *ASCL1* and *NEUROD1*, respectively (Supplementary Fig. 3c). In one cohort of 79 SCLC tumors^16^, significant elevation of *IGFBP5* (Fig. 3e) and *B4GALT1* (Fig. 3f) was found in ASCL1^High^ SCLC samples relative to ASCL1^Low^ samples (Fig. 3d). We also found the similar results in another cohort of 23 human SCLC tumors^33^ (Fig. 3g–i). Collectively, these data validated the physiological relevance of IGFBP5 and B4GALT1 as specific secreted protein markers for ASCL1^High^ NE-lung cancers.

### IGFBP5 is a secreted marker for ASCL1^High^ NE-lung cancer

**Fig. 4:**
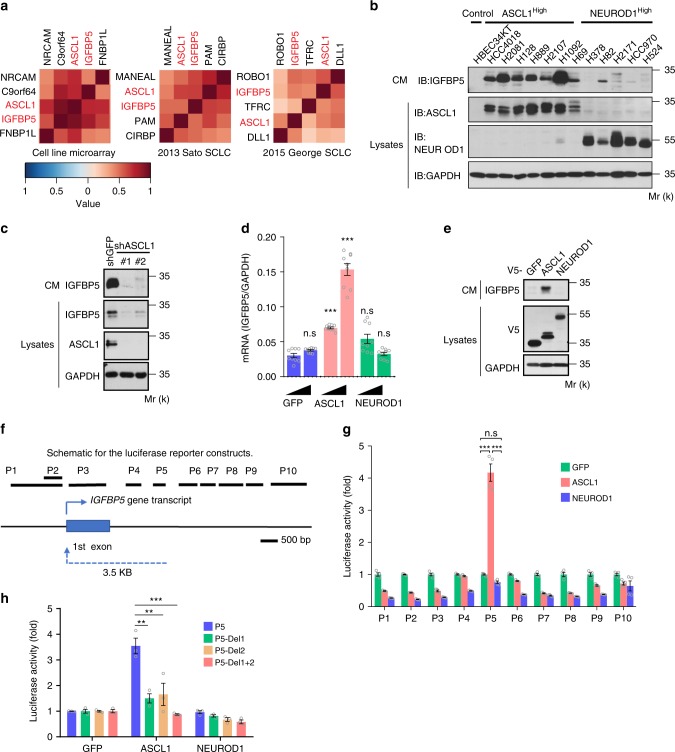
Identification of IGFBP5 as a direct transcriptional target of ASCL1. **a** Correlation heatmap illustrating clustered matrix of top four genes with the highest similarities with ASCL1 based on the expression profiles from three independent SCLC transcriptomic datasets. **b** Western blot of IGFBP5 in the CM, ASCL1, and NEUROD1 in the lysates of the indicated cell lines (HBEC34-KT and NE-lung cancer cell lines). Molecular weight is indicated as Mr (k). **c** Immunoblotting analyses of the IGFBP5 levels in the CM and lysates of H2081 cells transduced with the indicated shRNAs. **d**, **e** RT-PCR (*n* = 9, 3 independent experiments × 3 replicates) (**d**) and immunoblotting (**e**) analyses of IGFBP5 expression in HEK293T cells transfected with the indicated expression constructs. **f** Schematic for the various IGFBP5 luciferase reporter constructs. **g** Luciferase activity analysis of HEK293T cells transfected with the indicated IGFBP5 luciferase reporter constructs, together with V5-tagged GFP, ASCL1, or NEUROD1 (*n* = 4 replicates). **h** Luciferase activity of HEK293T cells transfected with the indicated deletion mutants of IGFBP5 luciferase reporter constructs, together with V5-tagged GFP, ASCL1, or NEUROD1 (*n* = 3 replicates). Unpaired two-tailed *t*-test. **P < 0.01, ***P < 0.001, n.s not significant. Error bars represent mean ± standard error of mean (s.e.m.). Source data are provided as a Source Data file

To further analyze the co-expression pattern between the AS/ND-LCSS and ASCL1/NEUORD1, we performed unsupervised hierarchical clustering on pairwise Pearson correlations for these genes in three different transcriptome datasets (SCLC cell line microarray^5^, 2013 Sato SCLC^33^, and 2015 George SCLC^16^). The results showed that *IGFBP5* was consistently found to be among the top four genes that best correlated with ASCL1 in all three transcriptome datasets (Fig. 4a, Supplementary Fig. 4a-c). Furthermore, we harvested the CM and cell lysates from a panel of lung cancer cell lines used in the TMT analysis, and performed immunoblotting analyses. Intriguingly, our results showed that IGFBP5 was highly and specifically secreted in the CM of the ASCL1^High^ NE-NSCLC and SCLC cells. In contrast, it was barely detectable in the CM from the NEUROD1^High^ SCLC cells or HBEC34-KT cells (Fig. 4b).

We also validated our results using an in vivo model of ASCL1^High^ SCLC. Most human ASCL1^High^ SCLC tumors harbor *TP53* and *Rb1* mutations. Genetically engineered mouse models with somatic deletion of *TP53* and *Rb1* in lung are able to recapitulate many clinical manifestations of ASCL1^High^ SCLC, including the extrapulmonary metastases^34,35^. The tumors from *Rb1/Tp53/Rbl2* triple-knockout (*TCKO*) mouse model express high levels of ASCL1. In addition, the histopathology of the metastatic mouse tumors also closely resembles human ASCL1^High^ SCLC^10^. Using an ELISA assay, we also found that IGFBP5 levels in the serum of mice with SCLC tumors were significantly higher than that in the control mice (Supplementary Fig. 4d). Taken together, these data showed the physiological relevance of IGFBP5 as a secreted biomarker for ASCL1^High^ SCLC in mouse models.

### *IGFBP5* is a transcriptional target of ASCL1

As ASCL1 is a transcription factor, we wondered if the robust correlation between the expression of *IGFBP5* and *ASCL1* could result from *IGFBP5* being a direct transcription target of ASCL1. To test this possibility, we knocked down ASCL1 in H2081 cells by using two independent shRNAs. Immunoblotting analysis indicated that depletion of ASCL1 markedly lowered IGFBP5 levels in both CM and lysates of H2081 cells (Fig. 4c). These data indicate that ASCL1 is necessary for IGFBP5 expression. To examine whether ASCL1 is sufficient to drive the expression of IGFBP5, we overexpressed ASCL1, NEUROD1, and GFP in HEK293T cells, and analyzed *IGFBP5* expression by real-time PCR. ASCL1 overexpression significantly increased *IGFBP5* mRNA levels in a dose-dependent manner, while expression of GFP or NEUROD1 had no effects on *IGFBP5* mRNA levels (Fig. 4d). We also harvested the CM and cell lysates from HEK293T cells transfected with these three plasmids. Immunoblotting analyses indicated that ASCL1, but not GFP or NEUROD1, strongly promoted the upregulation of IGFBP5 in the CM (Fig. 4e).

To examine whether IGFBP5 is a direct transcriptional target of ASCL1, we co-expressed a pLenti-V5-ASCL1 plasmid together with a series of pGL4 luciferase reporter constructs containing inserts representing different regions of the *IGFBP5* gene in HEK293T cells (Fig. 4f). We found that co-transfection of ASCL1 with one pGL4 construct (pGL4-P5-luc) that corresponded to a 300 bp (from +3.2 kb to +3.5 kb) fragment downstream of the *IGFBP5* transcription start site (TSS) led to a fourfold increase in luciferase activity (Fig. 4g). Consistent with these results, previous ChIP-seq data in an ASCL1^High^ cell line (H889) also showed that ASCL1 but not NEUROD1 binds to the +3325– +3453 region of the *IGFBP5* gene^10^ (Supplementary Fig. 4e). These data suggested that there could be potential ASCL1-responsive elements in this region of the *IGFBP5* gene. It was reported that ASCL1 binds to DNA sequences containing the E-box motif (CANNTG)^10,36^. We analyzed the DNA sequence of pGL4-P5-luc, and found two such E-box motifs (Supplementary Fig. 4f). We found that deletion of either E-box1 or E-box2 in P5 lowered the ability of ASCL1 to activate transcription through this region using the luciferase reporter assay (Fig. 4h). When both E-boxes were mutated, no activation of *IGFBP5* transcription was detected. These results suggest that these two E-box motifs are critical for ASCL1-dependent transcription regulation of *IGFBP5*. Taken together, these data support a model whereby *IGFBP5* is a direct transcriptional target of ASCL1.

### JQ-1 and IGF-1R inhibitor combination in ASCL1^High^ SCLC

It has been shown that ASCL1, the lineage-specific transcription factor for high-grade NE-lung cancer, is required for the survival of these tumors both in vitro^5,9^ and in the lung in vivo^10^. These findings point to targeting ASCL1 and/or its critical downstream genes may serve as a viable strategy for the treatment of ASCL1-dependent NE-lung cancers. The BET (bromodomain and extra-terminal) inhibitors have been shown to disrupt the interaction between BRD4 and the *ASCL1* enhancer, resulting in the downregulation of ASCL1, and subsequent growth inhibition of SCLC cells^37^. Because ASCL1 directly regulates the transcription of *IGFBP5*, we sought to determine the effect of BET inhibitors on IGFBP5 expression.

**Fig. 5:**
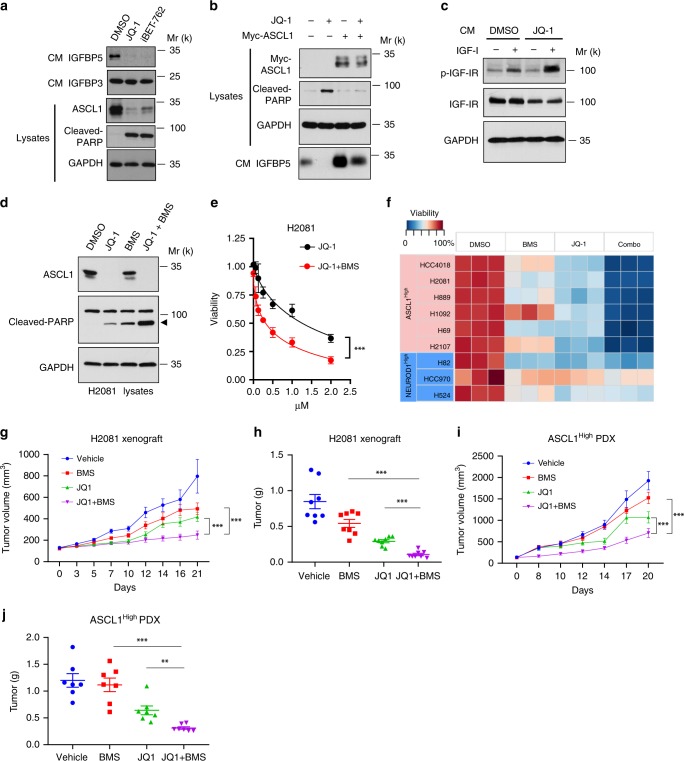
JQ-1 treatment sensitizes ASCL1^High^ SCLC cells to IGF-1R inhibitors. **a** Immunoblotting analyses of IGFBP5 and IGFBP3 levels in the CM of H2081 cells treated with DMSO, JQ-1 (1 µM for 48 h) and iBET-762 (2 µM for 48 h). **b** Immunoblotting analyses of indicated antibodies in H2081 cells transfected with empty vector or Myc-ASCL1 for 40 h, then treated with DMSO or JQ-1 (1 µM for 48 h). **c** Immunoblotting analysis of IGF-1-induced IGF-1R activation in H2081 treated as indicated. Serum-free CM was collected from H2081 cells treated with DMSO or JQ-1 (1 µM for 48 h). The CM samples were mixed with IGF-1 (20 ng ml^−1^), and then incubated with serum-starved H2081 cells (designated as “recipient cells”) for 10 min at 37 °C (IGF-1+). CM that was not mixed with IGF-1 is indicated as “IGF-1−”. For site-specific phosphorylation, p-IGF-1R(Tyr1135/1136) levels were analyzed. GAPDH served as the loading control. **d** Immunoblotting analysis of ASCL1 and PARP cleavage in H2081 cells treated with DMSO, JQ-1 (1 µM), BMS-754807 (1 µM), and JQ-1+BMS-754807 (1 µM each) for 48 h, respectively. **e** Cell viability analysis of H2081 cells treated with JQ-1 or JQ-1+BMS-754807 at the indicated concentrations for 72 h (*n* = 4). Unpaired two-tailed *t*-test. ***P < 0.001, n.s not significant. **f** Heatmap illustrating the cell viability analyses of the various NE-lung cancer cells treated with DMSO, JQ-1 (1 µM), BMS-754807 (1 µM), and combo (JQ-1 + BMS-754807 1 µM each) (72-h treatment). ASCL1^High^ and NEUROD1^High^ lines were marked as indicated. **g**, **i** Tumor sizes in an H2081 xenograft model (**g**, *n* = 9–10 per group) and an ASCL1^High^ PDX model (**i**, *n* = 10 per group) model. The mice were treated with the indicated compounds. Two-way analysis of variance (ANOVA), ***P < 0.001. **h**, **j** Final tumor weights measured from the H2081 (*n* = 8) (**h**) and PDX (*n* = 7) (**j**) models. Unpaired two-tailed *t*-test, **P < 0.01, ***P < 0.001. Error bars represent mean ± standard error of mean (s.e.m.). Source data are provided as a Source Data file

We treated H2081 cells with JQ-1, and then harvested the CM and cell lysates. Consistent with the previous findings^37^, immunoblotting experiments showed that ASCL1 expression was markedly reduced by JQ-1 treatment (Fig. 5a). Remarkably, IGFBP5 levels in the CM were also strongly decreased after the treatment of JQ-1 (Fig. 5a). In contrast, JQ-1 treatment had no effect on IGFBP3 abundances in the CM (Fig. 5a). Treatment with another BET inhibitor iBET-762 showed similar effects (Fig. 5a). Moreover, overexpression of a Myc-tagged ASCL1 could rescue JQ-1-induced cell apoptosis and IGFBP5 downregulation (Fig. 5b), indicating the regulation of IGFBP5 expression by JQ-1 is ASCL1-dependent.

Insulin-like growth factor-binding proteins (IGFBPs) are a family of secreted proteins that bind, with high affinity, to circulating IGF-1. We previously showed that by binding to IGF-1, IGFBP5 sequesters it from interacting with IGF-1R and potently blocks both the signaling and functional outputs of this growth factor^38^. We reasoned that JQ-1- treatment might release the cell autonomous inhibition of IGF-1 signaling by IGFBP5. This might drive the survival of the JQ-1-treated cells to be dependent on IGF-1 signaling, which, in turn, could confer their sensitivity to IGF-1R inhibitors under these ASCL1-repressed conditions. To test this hypothesis, we collected the CM from H2081 cells treated with either DMSO or JQ-1. Then the CM was mixed with IGF-1, which was used to stimulate IGF-1 signaling in the recipient cells. As shown in Fig. 5c, although IGF-1 treatment activates IGF-1R in the control group, the degree of IGF-1R activation is dramatically increased when cells were treated with IGF-1 mixed with the CM from JQ-1-treated cells. These data are consistent with a model whereby the presence of IGFBP5 in the media inhibits IGF-1 signaling, and ASCL1 downregulation releases this inhibitory mechanism, which then sensitizes these cells to IGF-1 stimulation.

To test whether the enhanced IGF-1R signaling promotes the survival of these JQ-1 treated cells, we evaluated the growth inhibitory effect of JQ-1, BMS-754807 (a potent inhibitor of IGF-1R), and the combination of JQ-1 with BMS-754807 on SCLC cell lines. Using cleaved PARP1 and cellular ATP levels as the readout, we found a dose-dependent, synergistic effect in ASCL1^High^ SCLC cells (H2081), when these cells were treated with both JQ-1 and BMS-754807 (Fig. 5d, e and Supplementary Fig. 5a,b). In contrast, JQ-1 treatment had no effect on the expression of NEUROD1 levels in NEUROD1^High^ SCLC cells (i.e., H524 cells) (Supplementary Fig. 5c). Accordingly, although H524 cells are partially sensitive to IGF-1R inhibitors, the combination of JQ-1 and BMS-754807 did not lead to enhanced killing of these cells (Supplementary Fig. 5d). Then we assessed the drug combination in an expanded panel of SCLC cell lines (Fig. 5f). Consistent with our previous findings, JQ-1/BMS-754807 combination treatment induced robust cell death in all tested ASCL1^High^ SCLC cells, but not NEUROD1^High^ cells (Fig. 5f). Taken together, these results suggest that bromodomain inhibitors decrease ASCL1 expression and downregulate its downstream target gene, *IGFBP5*. The release of this inhibitory loop results in enhanced IGF-1R signaling and sensitizes these ASCL1^High^ SCLC cells to IGF-1R inhibitors.

We next examined the in vivo anti-tumor efficacy of this combination treatment in the H2081 xenograft model. Consistent with our in vitro observations, monotherapy with either JQ-1 or BMS-754807 had modest effects on tumor growth (Fig. 5g, h) and cell apoptosis (Supplementary Fig. 5e). Importantly, JQ-1/ BMS-754807 combination treatment significantly inhibited tumor growth, potentially through enhanced cell death (Fig. 5g, h, Supplementary Fig. 5e). The therapeutic effects of JQ-1/BMS-754807 combination treatment were further demonstrated in a patient-derived xenograft model of ASCL1^High^ SCLC (Fig. 5i, j). Collectively, these data showed the clinical relevance of the combination therapeutic strategies for ASCL1^High^ SCLC.

## Discussion

During the process of oncogenic transformation, cancer cells not only adopt several cell autonomous hallmarks (e.g., deregulation of proliferative pathways and evasion of apoptosis), they also actively secrete a variety of biomolecules to communicate with and to engage their neighboring cells to create a specific tissue microenvironment^39^. Protein secretion is particularly relevant to the etiology of SCLC, and accumulated evidence has pointed to the indispensable role played by the deregulated secretome in almost every aspect of the oncogenesis of pulmonary NE tumors^1^. Specifically, it is thought that SCLC originates from the transformation of the pulmonary NE cells (PNECs). The function of PNECs is to serve as airway chemoreceptors, and they respond to stimuli (e.g., hypoxia) by degranulation and exocytosis of a large variety of bioactive molecules, including amines and neuropeptides/proteins. Many of these proteins with growth factor-like properties are linked to not only normal lung development, but also tumorigenesis of SCLC^40,41^. Furthermore, excessive secretion of neuropeptides and hormones by pulmonary NE tumors could lead to many paraneoplastic syndromes, including the syndrome of inappropriate anti diuretic hormone secretion (SIADH) and Cushing syndrome^42^. If improperly treated, these paraneoplastic syndromes could also lead to considerable morbidity and mortality. A comprehensive characterization of the secretome associated with SCLC may therefore facilitate the understanding of the biology of pulmonary NE tumors. Equally important, secreted proteins may represent a class of systemic biomarkers for early diagnosis and monitoring of drug response for this devastating disease. In this study, we performed large-scale, unbiased quantitative proteomic analyses of the secretome from cells derived from both the ASCL1^High^ and NEUROD1^High^ subtype of pulmonary NE tumors. It is important to note that ASCL1 and NEUROD1 have been reported to be overexpressed in ~70% and ~10–20% of the SCLC cases, respectively^1,11,30,31,43^ ASCL1 is required to establish the lineage of pulmonary NE cells and is necessary for the continued survival of SCLCs and NE-NSCLC in cell cultures. In ASCL1^High^ SCLC and NE-NSCLC, knockdown of ASCL1 induces cell death, suggesting the role of ASCL1 for tumor cell survival^5^. In GEMM mouse models of SCLC, it was found that ASCL1 is present in mouse pulmonary NE cells, and is required for SCLC tumor formation^10^. More importantly, ASCL1 is expressed in most human SCLC tumors and cell lines and its expression is tightly linked to NE differentiation, highlighting the importance of identifying the secreted biomarker for ASCL1^High^ SCLC.

Comprehensive analysis of secreted proteins, however, is challenged by a number of technical difficulties. Many extracellular proteins are signaling molecules that are present at low levels. These proteins could be easily masked by more abundant proteins, which results in the extraordinary dynamic range (defined by the ratio between the most and least abundant protein in a sample) of the secretome. We recently developed a high sensitivity mass spectrometry platform that is tailored to the analysis of the secretome. This platform combines multi-dimensional HPLC with high sensitivity mass spectrometry to allow comprehensive analysis of proteins secreted from NSCLC cells^23^. In the current study, we were able to identify and quantify 1626 proteins in the CM from 13 cell lines representing normal lung and NE-pulmonary tumor. The basic helix–loop–helix (bHLH) transcription factor ASCL1 is considered a master regulator for a majority of NE-lung cancers, while NEUROD1 signifies a smaller subset with intermediate neuroendocrine characteristics^30^. Thus, ASCL1 and NEUROD1 expression has been used to divide NE-lung cancers into ASCL1^High^, NEUROD1^High^, or double-negative subtypes. The transcriptome of NE-lung cancers driven by ASCL1 or NEUROD1 has been well studied^5,10,16,31^. Our study comprehensively analyzed the secretome of various SCLC cell lines including both ASCL1^High^ and NEUROD1^High^ subtypes, suggesting the lineage-specific transcription factors ASCL1^High^ and NEUROD1^High^ also drive distinct secretome. Appreciation of the heterogeneity of SCLC tumor subtypes is driving efforts to define subtype-specific markers to select patient populations for clinical trials and precision treatment. This SCLC secretome dataset could be a resource for specific biomarkers for these two major subtypes of NE-lung cancers.

To prioritize our candidates, a combination of statistical and biological filtering was used, including: presence in the HUPO^27^, the differential protein level in these two subtypes of NE-lung cancer secretome, and unsupervised clustering analysis using previous published transcriptome datasets. After the comprehensive analysis, two genes (*IGFBP5* and *B4GALT1*) and one gene (*ANXA6*) were found consistently clustered with *ASCL1* and *NEUROD1*, respectively. Using a series of biochemical experiments, we demonstrated that IGFBP5 is a direct transcriptional target of ASCL1. On the contrary, *B4GALT1* expression is not regulated by ASCL1^10^, suggesting ASCL1 regulates the SCLC-specific secretome through both transcription-depend and -independent mechanisms.

Because ASCL1 is necessary for the survival and proliferation of ASCL1^High^ NE-lung cancer, targeting ASCL1 is a promising strategy to kill ASCL1^High^ SCLC. It has been reported ASCL1^High^ SCLC cells are highly sensitive to BET inhibitors (e.g., JQ-1). These compounds inhibit ASCL1 expression by disrupting the interaction between BRD4 and the ASCL1 enhancer^37^. In keeping with these results, we found that JQ-1 decreased the expression of ASCL1, but not NEUROD1. Accordingly, it also decreased IGFBP5 in the CM of ASCL1^High^ SCLC cell lines. Recently we found that IGFBP5 is a secreted protein that binds to IGF-1 and prevents it from engaging with IGF-1R. In doing so, it potently blocks both the signaling and functional outputs of IGF-1^38^. Based on these findings^11,38^, our results raise an intriguing hypothesis that, despite the pro-survival effects of ASCL1, it also orchestrates an IGFBP5-depdenent inhibitory mechanism to restrain IGF-1R signaling. Reduced IGFBP5 secretion after JQ-1 treatment could result in enhanced IGF-1 signaling, serving as a compensatory mechanism for maintaining cell survival and proliferation under these ASCL1-suppressed conditions. We therefore tested the combination treatment in various SCLC cell lines and found the synergistic effect of JQ-1 and BMS-754807 (a potent inhibitor of IGF-1R), specifically in ASCL1^High^ SCLC cells. Moreover, we also validated this combination treatment using two in vivo models (i.e., xenograft and PDX models). Such efforts might pave the way for the further evaluation of such combination therapies for ASCL1^High^ NE-lung cancer patients.

It has been recently demonstrated that POU2F3 is a transcription factor that signifies a group of SCLC tumors that lack the expression of traditional NE markers^11^. By performing a kinase-focused CRISPR screen, it was found that the survival of these POU2F3-positive SCLC cells is dependent on IGF-1R or its downstream PI3K signaling pathways. POU2F3^High^ lines are consistently more sensitive to IGF-1R inhibitors than NEUROD1^High^ or ASCL1^High^ SCLC lines. Interestingly, compared to ASCL1^High^ SCLC cells, IGFBP5 expression is much lower in these POU2F3^High^ cells, which might explain their addiction to IGF-1R signaling, and therefore intrinsic sensitivity to IGF-1R inhibitors. Despite the different SCLC subtypes and contexts, these data point to a central role of IGF-1R signaling in regulating the survival and proliferation of ASCL1^High^ and POU2F3^High^ SCLC cells, which warrants further studies to explore therapeutic strategies targeting this important pathway in these two subtypes of SCLC.

**Fig. 6:**
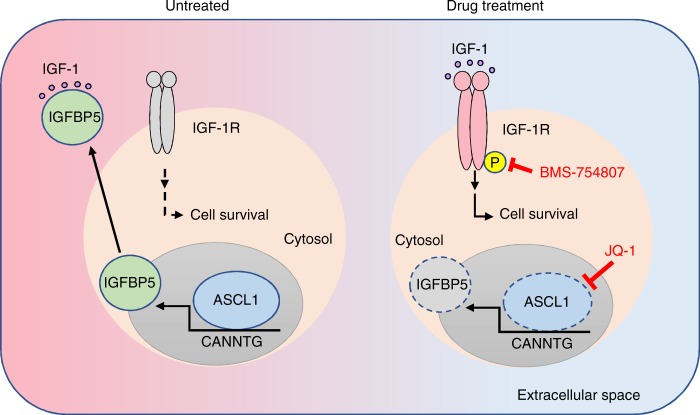
ASCL1 orchestrates an IGFBP5-dependent mechanism to suppress IGF-1 signaling. Under basal conditions, ASCL1 promotes the transcription and expression of IGFBP5, which accumulates in the extracellular space. In doing so, IGFBP5 inhibits IGF-1 from binding to its cognate receptor (IGF-1R). JQ-1-treatment leads to the downregulation of ASCL1 and subsequently, IGFBP5. This de-represses IGF-1 signaling and renders the cells more sensitive to IGF-1R inhibitors. This model suggests that a combination of JQ-1 and IGF-1R inhibitors might be more effective in killing ASCL1^High^ NE-lung cancers

In summary, we performed comprehensive mass spectrometric analyses of the secretome of ASCL1^High^ and NEUROD1^High^ pulmonary NE tumor cells. Compared to the NSCLC secretome, these NE-lung cancer cells secrete a number of proteins that are uniquely linked to the biology of this very interesting group of lung cancer. In particular, we identified IGFBP5 as a secreted protein that is specifically regulated by the lineage-specific transcription factor ASCL1. Furthermore, we showed that JQ-1-mediated ASCL1 suppression paradoxically leads to the de-repression of IGF-1R signaling, which, in turn, renders these cells sensitive to combination treatment of JQ-1 and IGF-1R inhibitors (Fig. 6). Besides the identification of IGFBP5 as a potential biomarker for ASCL1^High^ SCLC, and its role as a mediator of its adaptive response to bromodomain inhibitors, we expect that the datasets contain many previously unrecognized secreted targets of ASCL1 and NEUROD1 that will serve as an invaluable resource for future biomarker discovery, as well as for hypothesis-driven research that helps dissect the complex molecular underpinnings that drive the oncogenesis of pulmonary NE tumors.

## Methods

### Materials

IGF-1R (#3027, CST, dilution 1:1000), p-IGF-1R (Y1135/1136) (#3024, CST, dilution 1:1000), IGFBP5 (p-19, Santa Cruz, dilution 1:750), V5 (A190-120A, Bethyl Laboratories, Inc., dilution 1:2000), Myc-Tag (9B11) (#2276, CST, dilution 1:2000), GAPDH (sc-32233, Santa Cruz, dilution 1:5000), NEUROD1 (ab60704, Abcam, dilution 1:1000), IGFBP3 (09–180, EMD Millipore, dilution 1:1000), Cleaved Caspase-3 (Asp175) (#9661s, CST, dilution 1:200), cleaved PARP (Asp214) (#9546s, CST, dilution 1:1000) were obtained from commercial sources. ASCL1 (J.E.J. lab TX518, dilution 1:5000) antibody was generated as described^44^. Keratinocyte-SFM, Bovine Pituitary Extract (BPE) and Epidermal Growth Factor (EGF) were purchased from Life Technologies. RPMI-1640 and Fetal Bovine Serum (FBS) were purchased from Sigma-Aldrich. LDH cytotoxicity assay kit was obtained from Pierce (Life technologies).

### Plasmids

The cDNAs for GFP, human ASCL1 and NEUROD1 were obtained from Invitrogen, amplified by PCR, and cloned into the pLenti6.3/V5-DEST destination vector using Gateway Recombination (Thermo). Lentiviral plasmids (Δ8.9 and VSVG) were kind gifts from A. Kung (Dana Farber Cancer Institute, USA) and D. Baltimore (California Institute of Technology, USA). The sequences for those plasmids are shown in Supplementary Table 2.

### Mammalian lentiviral shRNAs

Lentiviral small hairpin RNA (shRNA) targeting *ASCL1* in pLKO.1 expression vectors (Clone ID: NM_004316.1-1023s1c1 and NM_004316.3-955s21c1) were obtained from Sigma (The shRNA sequences are listed in Supplementary Table 2). To generate the lentiviruses, shRNA plasmids were co-transfected into HEK293TD cells along with packaging (Δ8.9) and envelope (VSVG) expression plasmids using the Lipofectamine 2000 reagent (Invitrogen). Two days after transfection, viral supernatants were collected and filtered. Recipient cells were infected in the presence of a serum-containing medium supplemented with 8 μg ml^−1^ Polybrene. Forty eight hours after infection, cells were used for the indicated experiments. Knockdown efficiencies were examined by immunoblot assays using the antibodies against ASCL1.

### Cell culture

All lung cell lines used in this study were obtained from the Hamon Cancer Center Collection (University of Texas Southwestern Medical Center, Dallas, TX). HBEC34-KT cells were cultured in Keratinocyte-SFM (GIBCO) supplemented with BPE and EGF (GIBCO) at 37 °C in 5% CO_2_. H2081, HCC4018, H889, H1092, H69, H2107, H128, H378, H82, H2171, HCC970, and H524 cells were cultured in RPMI-1640 (Sigma) with 10% FBS (Sigma) at 37 °C in 5% CO_2_. All cell lines have been DNA fingerprinted using the PowerPlex 1.2 kit (Promega) and are found to be mycoplasma free using the e-Myco kit (Boca Scientific).

### Western blotting analysis

Cells were harvested and lysed in the SDS lysis buffer (1% SDS, 10 mM HEPES, pH 7.0, 2 mM MgCl_2_, universal nuclease 20 U ml^−1^). Total cellular protein concentration was measured by the BCA assay kit (Thermo Fisher Scientific). Equal amounts (20 μg) of the protein samples were subjected to 10% SDS-PAGE and transferred to nitrocellulose blotting membranes (GE Healthcare). The membranes were incubated in the blocking buffer for 60 min at room temperature to reduce non-specific binding of the primary antibodies. The membranes were then blotted with the primary antibodies overnight at 4 °C, after which, the membranes were washed three times in TBST (10 min per wash on a rocking platform) which was followed by incubation with the secondary antibody for 1 h at room temperature. Proteins were developed using enhanced chemiluminescence exposed on an autoradiograph film.

### Secretome sample preparation for mass spectrometric analysis

For HBEC34-KT cells, 2.5 million cells were plated in Keratinocyte-SFM (GIBCO) supplemented with BPE and EGF for 16 h. The next day cells were washed with PBS twice and the medium was changed to Keratinocyte-SFM without BPE or EGF. Two dishes of cells (15 cm) were starved in SFM at 37oC in 5% CO2 for 24 h, and the CM was collected and mixed with SDS (1% final concentration). The CM was then centrifuged (300×*g*, 3 min), filtered (0.45 μM Polythersulfone Membrane, GE healthcare) and lyophilized. Secreted proteins were extracted by methanol/chloroform precipitation (methanol: chloroform: H_2_O: sample = 4:1:3:1) and were subsequently washed by ice-cold methanol. Protein pellets were re-solubilized in 2 ml freshly prepared 8 M urea buffer (containing 50 mM Tris, 10 mM EDTA, pH 7.5) and concentrations were measured by the BCA assay (Thermo Scientific). Proteins were reduced (2 mM DTT for 15 min at RT) and alkylated (30 mM iodoacetamide for 30 min at RT). Proteins were then digested by Lys-C at a 1:100 (w/w) enzyme/protein ratio for 2 h, followed by trypsin digestion at a 1:100 (w/w) enzyme/protein ratio overnight at RT. Peptides were desalted by using Oasis HLB solid-phase extraction (SPE) cartridges (Waters)^45^.

Desalted peptides were resuspended in 200 mM HEPES pH 8.5. Approximately 100 μg peptides were reacted with the amine-based TMT six-plex reagents (Thermo Fisher) for 1 h at room temperature (i.e., channel 126 and 127 for HBEC34-KT, 128 and 129 for HCC4018, and then 130 and 131 for H2081 cells). Hydroxylamine solution was added to quench the reaction and the labeled peptide samples were combined. The TMT sample was lyophilized and reconstituted in 400 μl buffer A (5 mM KH_2_PO_4_, pH 2.65, 30% acetonitrile). It was then centrifuged at 10,000×*g* for 3 min using spin-X centrifuge tube filters (Corning) prior to loading onto a SCX column (PolySULFOETHYL A^TM^, 200 mm × 4.6 mm, 5 μm particle size, 200 Ǻ pore size, PolyLC). Peptides were fractioned by SCX-HPLC at a flow rate of 1 ml min^−1^. Gradient was developed from 0 to 21% buffer B (5 mM KH_2_PO_4_, pH 2.65, 30% acetonitrile, 350 mM KCl)^23,46^. Seventeen fractions were collected, which were lyophilized, desalted, and analyzed by LC–MS/MS.

### LC–MS/MS analysis

The TMT sample was analyzed by LC–MS/MS on an LTQ Velos Pro Orbitrap mass spectrometer (Thermo, San Jose, CA) using a top ten HCD (higher-energy collisional dissociation) method (collision energy set at 34 eV). Orbitrap resolution for precursor and fragment ions was set to be 60,000 and 7000, respectively. MS/MS spectra were searched against a composite database of human protein sequences (Uniprot) and their reversed complement using the Sequest algorithm (Ver28) embedded in an in-house-developed software suite^47^. MS1 and MS2 mass tolerance was set to be 50 ppm and 0.05 Da, respectively. Search parameters allowed for full tryptic peptides (2 missed cleavage sites) with a static modification of 57.02146, 229.16293, and 229.16293 Da for Cys, Lys, and peptide N terminus, respectively. A dynamic modification of oxidation (15.99491 Da) was considered for Met. Default settings were used for min and max peptide lengths. Search results were filtered to include < 1% matches (both peptide and protein level filtering) to the reverse database by the linear discriminator function using parameters including Xcorr, dCN, missed cleavage, charge state (exclude 1 + peptides), mass accuracy, peptide length, and fraction of ions matched to MS/MS spectra. Peptide quantification was performed by using the CoreQuant algorithm implemented in an in-house-developed software suite^48^.

The labeling scheme for the TMT experiments is as follows: TMT set 1: 126: HBEC34-KT replicate 1; 127: HBEC34-KT replicate 2; 128: H4018 replicate 1; 129: H4018 replicate 2; 130: H2081 replicate 1; 131: H2081 replicate 2; TMT set 2: 126: HBEC34-KT; 127: H889; 128: H1092; 129: H69; 130: H2107; 131: H128; TMT set 3: 126: HBEC34-KT; 127: H378; 128: H82; 129: H2171; 130: HCC970; 131: H524. For TMT quantification, a 0.03 Th window was scanned around the theoretical *m*/*z* of each reporter ion (126: 126.127726; 127: 127.124761; 128: 128.134436; 129: 129.131471; 130: 130.141145; 131: 131.138180) to detect the presence of these ions. The maximum intensity of each ion is extracted, and the SN value of each protein is calculated by summing the reporter ion counts (SN) across all identified peptides. Because a same amount of peptides was used for each TMT channel, the total reporter ion intensity of each channel was summed across all quantified proteins, and was then normalized and reported.

### Protein–protein interaction network analysis

A protein interaction network was generated using the STRING database (Version 11.0)^29^. The interaction network of NE-LCSS genes was generated with a required confidence score of 0.4.

### Unsupervised relative expression based clustering

The signal-to-noise ratio (SN) of a protein was determined by summing the corresponding values for its individual peptides. These protein SN values were further processed and compared to the control group. H2081 and HCC4018 were compared to HBEC34-KT, while H2073 and H1993 were compared to HBEC3-KT^23^. These values were then transformed to a Log2 base (i.e., *Y* = log_2_ (*X*)). The subsequent hierarchical clustering was conducted using the complete agglomeration method of hclust as implemented in the heatmap2 R package. Selected proteins were analyzed by GO using the DAVID database (https://david.ncifcrf.gov/).

Unsupervised clustering was performed with the lung cancer cell line microarray data^5^, human SCLC tumor microarray data^33^, and RNA-seq data of 69 SCLC cases for which matching genome sequencing data was available^16^. For RNA-seq data, as expression values are following log-normal distribution, we transformed the raw FPKM value of each transcript by log2(1 + FPKM). Using the determined list of transcripts/genes, the subsequent hierarchical clustering was conducted in the heatmap2 R package.

### Luciferase reporter assays

To explore the potential ASCL1 binding sites in the *IGFBP5* gene, fragments of *IGFBP5* that are upstream and downstream of the genomic transcription start site were amplified and subcloned into the pGL4.42 (luc2P/HRE/Hygro) vector (Promega) using the XhoI and KpnI sites. Ten reporter vectors were generated with this strategy, pGL4.P1 (P1, −1.5 kb ∼−50 bp), pGL4.P2 (P2, −0.6 kb ∼−50 bp), pGL4.P3 (P3, +0.1 kb ∼+0.9 kb), pGL4.P4 (P4, +2.9 kb ∼+3.2 kb), pGL4.P5 (P5, +3.2 kb ~+3.5 kb), pGL4.P6 (P6, +6 kb ~+6.6 kb), pGL4.P7 (P7, +6.6 kb ~+7 kb), pGL4.P8 (P8, +7.2 kb ~+7.9 kb), pGL4.P9 (P9, +7.9 kb ~+8.4 kb) and pGL4.P10 (P10, +9 kb ~+10 kb). The E-box-deficient pGL4.42 mutants reporters were generated using the QuikChange site-directed mutagenesis kit (Stratagene). The sequences of the primers are listed in Supplementary Table 3. To perform the luciferase reporter assay, these pGL4 reporter vectors were co-transfected with pLenti-V5-ASCL1, pLenti-V5-NEUROD1 or pLenti-V5-GFP into HEK293T cells. The pGL4–hRluc expressing the Renilla reniformis luciferase under the TK promoter was used as the internal control in each experiment. Forty eight hours after transfection, cells lysates were collected and were used to determine the luciferase activity using the protocol from Promega. Experiments were performed in triplicate.

### IGFBP5 ELISA

The concentration of IGFBP5 in mouse sera was determined by an enzyme-linked immunosorbent assay according to the manufacturer’s instructions (DY578, R&D Systems). A 96-well plate (Corning Costar) was coated overnight at room temperature with a mouse IGFBP5 capture antibody. Triplicates of mouse IGFBP5 standards and serum from control mice and mice with SCLC tumors were then added to the plate, followed by incubation for 2 h at room temperature. After three washes, a biotinylated mouse IGFBP5 detection antibody was added to the plate, followed by incubation for 2 h at room temperature. Then the streptavidin-HRP antibody was added into each well, followed by 20 min incubation at room temperature. The amount of bound avidin was then assessed with TMB peroxidase that was acidified by 2 N H_2_SO_4_. The absorbance of each well at 450 nm was then measured with a spectrophotometric plate reader (BioTek).

### Cell viability assays

Three-day dose-dependent cell viability assays were carried out by plating 2000 cells per well of H2081, H69, or H524, respectively, into white transparent-bottom 96-well plates. On the same day, the cells were treated with JQ-1 or the combination of JQ-1 and BMS-754807 across a 6-dose range from 62.5 nM to 2 μM. For validation experiments, 2000–5000 cells (per well) of HCC4018, H2081, H889, H1092, H69, H2107, H82, HCC970, or H524 were plated into white transparent-bottom 96-well plates, and were then treated with DMSO, 1 μM JQ-1, 1 μM BMS-754807, or JQ-1 + BMS-754807 (1 μM each). After 72 h of drug treatment, cell viability was measured using the CellTiter-Glo assay (Promega).

### In vivo drug treatment experiments

The BET inhibitor JQ-1 and IGF-1R inhibitor BMS-754807 were purchased from Selleck Chemicals. Compounds were dissolved in 2% DMSO + 30% PEG 300 + 5% Tween 80 + ddH_2_O. Tumors were engrafted in NSG (NOD-SCID) mice (The Jackson Laboratory) by subcutaneous injection of 1 × 10^6^ cells in RPMI-1640 medium supplemented with 50% Matrigel (BD Biosciences, cat. no. 354234). Ten days after the injection, animals were assigned randomly to control and various treatment groups (*n* *=* 9–10 for each group). Tumor bearing mice were intraperitoneal injected with: (1) Vehicle, 2% DMSO + 30% PEG 300 + 5% Tween 80 + ddH_2_O; (2) JQ-1, 25 mg kg^−1^ day^−1^; (3) BMS-754807, 3.125 mg kg^−1^ day^−1^; (4) Combination of 25 mg kg^−1^ day^−1^ JQ-1 and 3.125 mg kg^−1^ day^−1^ BMS-754807. The mice were treated every other day. Tumors were measured with an external caliper, and the volume was calculated as (4*π*/3) × (width/2)^2^ × (length/2).

Statistical testing of the resulting data was conducted using the GraphPad Prism software (v8). To examine significance in xenograft between two groups, a two-way ANOVA was applied to compare the treated versus control groups, and a follow-up Tukey’s post hoc test was used for the various comparisons in the experiments. All animal procedures were reviewed and approved by the institutional animal care and use committee (IACUC) at UT Southwestern medical center. All animal studies were performed in compliance with the IACUC’s guidelines at UT Southwestern medical center.

### Immunohistochemistry

Resected xenograft tumors were fixed in neutral-buffered formalin (Sigma, F8775) for 48 h before processing to paraffin. Paraffin blocks were sectioned to a thickness of 4 μm. Immunohistochemical analysis was performed using a rabbit-anti-Cleaved Caspase-3 (Asp175) (1:200, #9661 s, CST) antibody with overnight incubation at 4 °C. The antigen in tissue sections was detected with the Mouse/Rabbit Specific HRP/DAB (ABC) Detection IHC Kit (Abcam, ab64264). Briefly, the specific antibody is located by a biotin-conjugated secondary antibody, followed by the addition of a streptavidin-enzyme conjugate and then visualized with the substrate-chromogen. Tissue samples were then imaged on a Keyence All-in-One microscope at ×40 with three fields per tissue slice.

### Statistical analysis

Hit calling is done by applying moderated *t*-test from R limma package 3.34.8^32^. The adjusted *P*-values were then calculated by using limma in R v3.2.3 statistical environment. All of the other statistical analyses (unpaired, two-tailed *t*-tests, and two-way ANOVA) were performed using the GraphPad Prism software (v8). Data were derived from the average of three biological replicate experiments and calculated as mean ± SEM. *P < 0.5, **P < 0.01, ***P < 0.001, n.s not significant.

### Reporting Summary

Further information on research design is available in the Nature Research Reporting Summary linked to this article.

## Supplementary information

Supplementary Information

Description of Additional Supplementary Files

Supplementary Data 1

Supplementary Data 2

Supplementary Data 3

Supplementary Data 4

Reporting Summary

Source Data

## Data Availability

The mass spectrometry data have been deposited to the ProteomeXchange Consortium (https://www.ebi.ac.uk/pride/archive/) via the PRIDE partner repository with the dataset identifiers: PXD013298 and PXD013267. Microarray, RNA-seq, and ChIP-seq data for human patient samples and cell lines were obtained from published literature^5,10,16,33^. The gene expression microarray data from the lung cancer cell lines were downloaded from the Gene Expression Omnibus (https://www.ncbi.nlm.nih.gov/geo/) with accession number GSE32036. The gene expression microarray data from the human SCLC tumor tissue was downloaded from GEO with accession numbers GSE43346. In this dataset, the Affymetrix GeneChip Human Genome U133 plus 2.0 oligonucleotide array data were analyzed using the Affymetrix GeneChip Operating Software v1.3 by MAS5 algorithms, to obtain signal value for each probeset. ChIP-seq libraries were sequenced on an Illumina High-Seq 2000 or Illumina GAIIx (GSE69398). The source data underlying Figs. 2b, 3a, 3d–i, 4d, 4g, 4h, 5e, 5f, 5g–i and Supplementary Figs. 1d, 1f, 2c, 4d, 5b and 5d are provided as a Source Data file. Fully uncropped versions of all gels and blots are shown in Supplementary Fig. 6. A reporting summary for this Article is available as a Supplementary Information file. Computer code and all the other data supporting the findings of this study are available from the corresponding author upon request.
