# Supplementary material for: Subtype-specific secretomic characterization of pulmonary neuroendocrine tumor cells
Source: Nat Commun. 2019 Jul 19;10:3201. doi: 10.1038/s41467-019-11153-5 (PMC6642156; doi:10.1038/s41467-019-11153-5)
Supplement: Supplementary file 1 — Supplementary Information [file 41467_2019_11153_MOESM1_ESM.pdf]

Supplementary Information

Subtype-Specific Secretomic Characterization of Pulmonary Neuroendocrine Tumors

Wang et al.

This PDF file includes:

Supplementary Figures 1-6

Supplementary Tables 1-3

Supplementary References

Supplementary Figures and Figure Legends

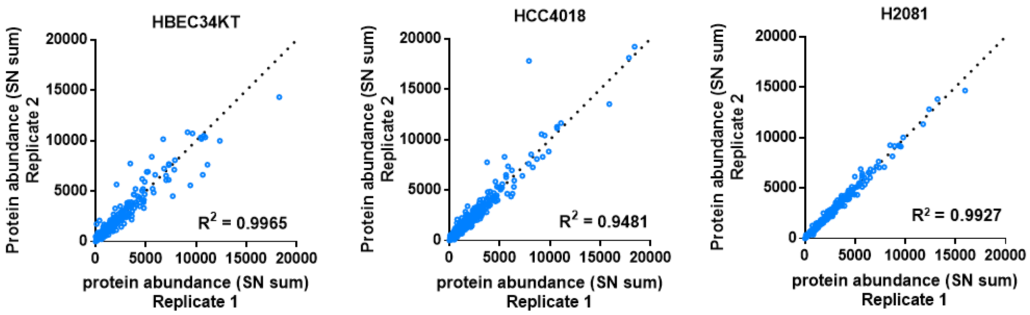

d

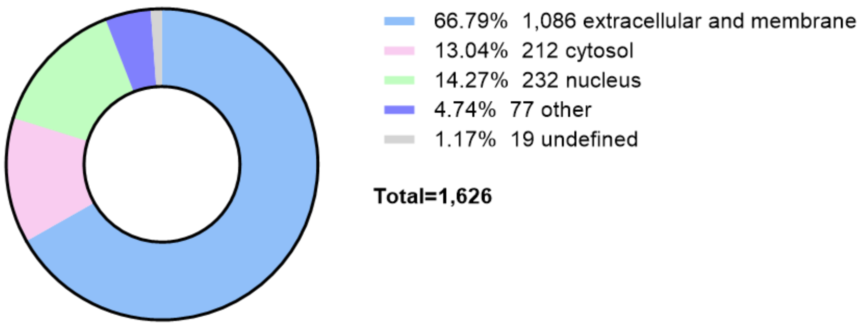

e

GO terms	protein count	%	P Value
extracellular exosome	841	52.33354	4.72E-300
cytoplasm	735	45.7374	3.87E-55
cytosol	668	41.56814	2.47E-120
membrane	418	26.0112	4.07E-60
extracellular space	262	16.30367	9.67E-38
focal adhesion	170	10.57872	9.30E-77
cell-cell adherens junction	156	9.70753	2.04E-78
extracellular matrix	128	7.965152	3.85E-57

f

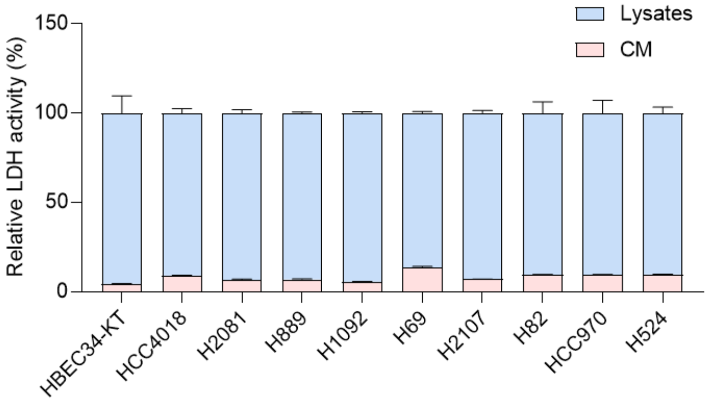

Supplementary Figure. 1, related to Figure. 1 Quantitative mass spectrometric analysis of the NE lung cancer secretome

a-c Reproducibility of the TMT experiments. Two biological replicate experiments were performed for HBEC34-KT (**a**), HCC4018 (**b**) and H2081 (**c**). **d** The exclusive protein

counts for each highly represented GO cellular component term. **e** Summary table for each highly represented GO cellular component term. **f** Summary metrics for the LDH activity assay of the CM (pink) and lysates (light blue) of the indicated cells starved in FBS free medium for 24 hours.

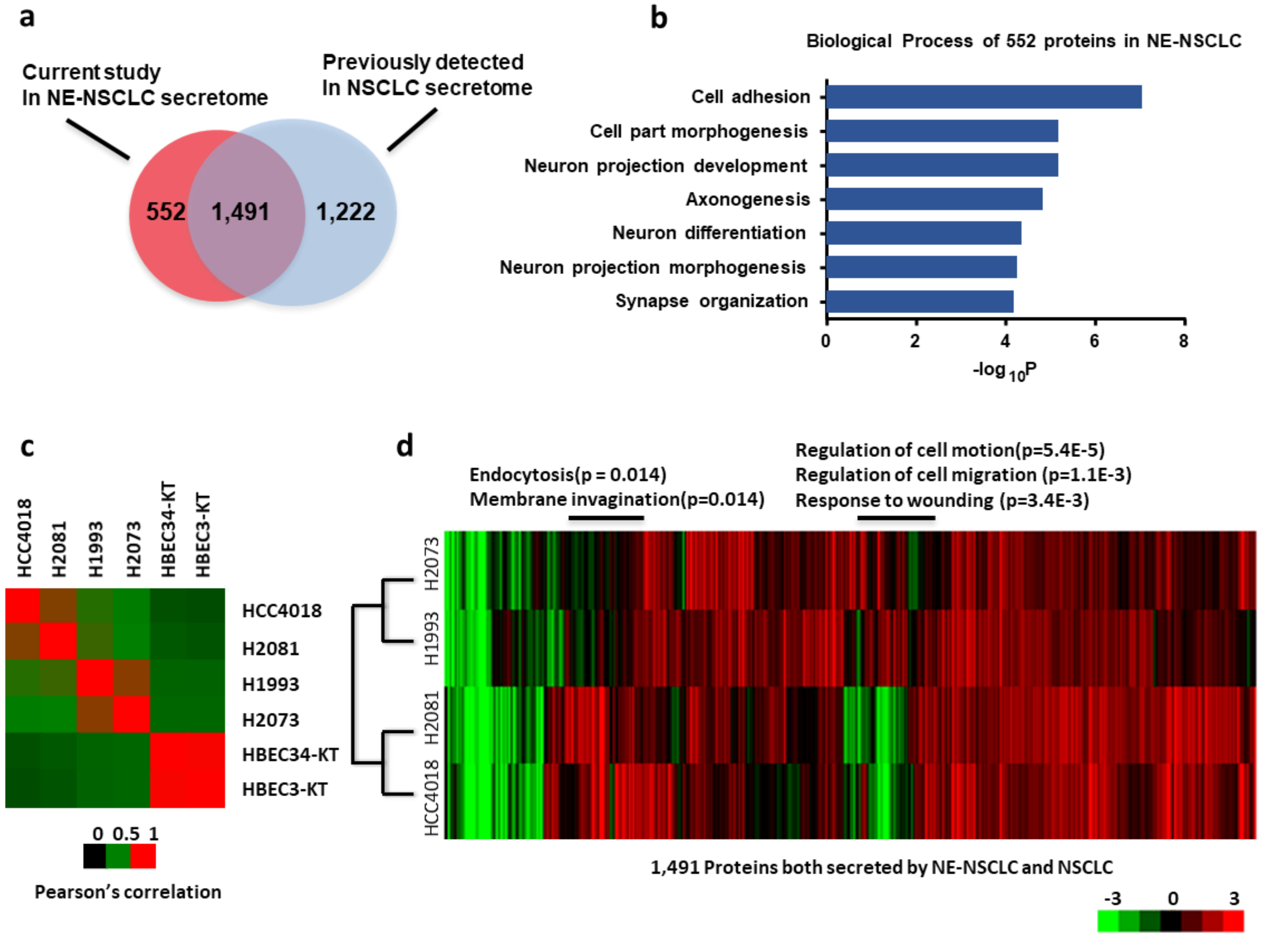

Supplementary Figure. 2, related to Figure. 2 Comparison of the secretome between high-grade NE NSCLC and classic NSCLC

a Venn diagram showing the proteins that were commonly identified in the CM from NE-lung cancer (current study) vs. NSCLC[1]. **b** Gene Ontology (Biological Process) analysis of the 552 proteins that were uniquely identified in the CM from NE-lung cancer. **c** Pearson's correlation (based on protein expression) of the 1,491 proteins that were commonly identified in all six cell lines (HBEC3-KT, HBEC34-KT, H1993, H2073, HCC4018 and H2081). **d** Unsupervised hierarchical clustering analysis of protein

expression in the CM from the NSCLC and NE-lung cancer lines (normalized to HBEC cells, shown as log2-transformed ratios). Representative proteins that were differentially expressed present in NSCLC or NE-lung cancer cell lines were subjected to GO analysis.

a

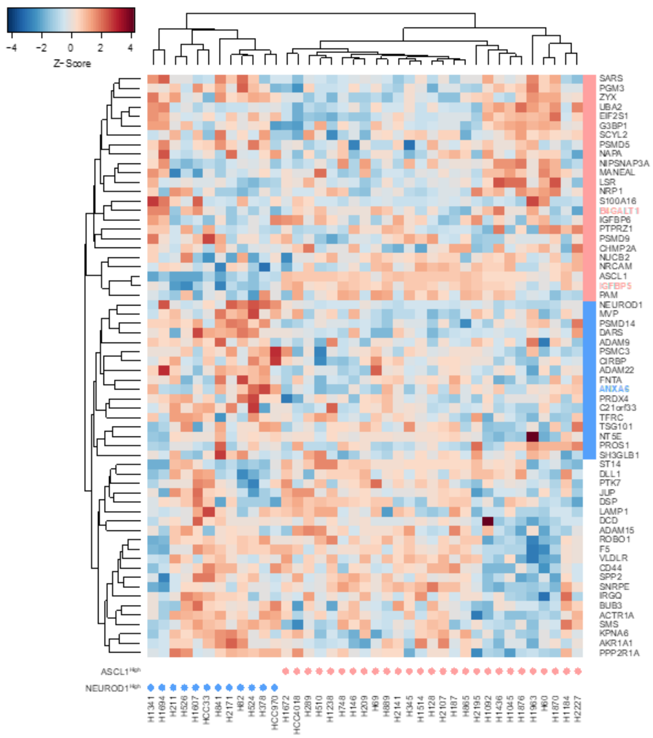

b

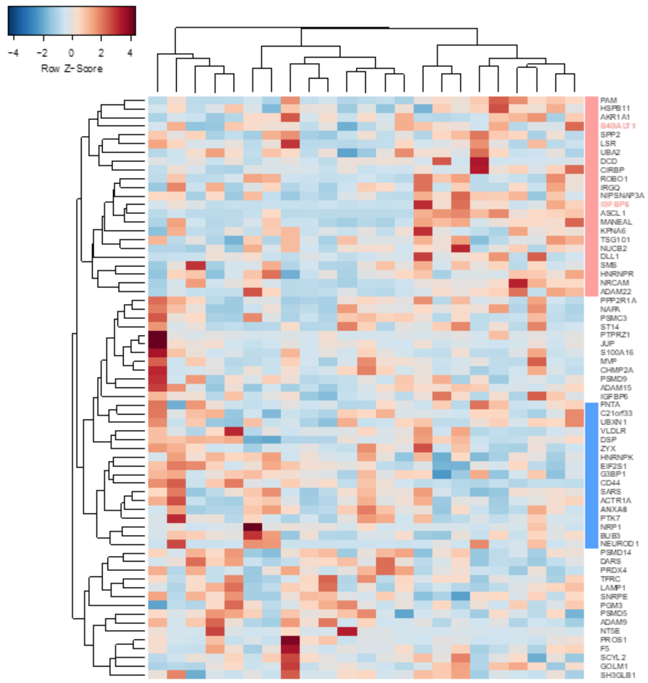

c

Gene Symbol	cell line microarray	Geroge	Sato
ST14		y	y
SNRPE			
IGFBP5	y	y	y
DLL1		y	y
DCD			
PTPRZ1	y		
NRP1			
HSPB11			
LSR		y	y
CD44			
LAMP1		y	y
PAM	y		
PROS1			
ADAM22			
B4GALT1	y	y	y
ADAM9			
SCYL2			
VLDLR		y	y
DSP		y	y
SPP2		y	y
GOLM1			
F5			
JUP		y	y
ADAM15		y	y
KPNA6			
IGFBP6		y	y
CIRBP			
NRCAM	y		
NAPA			
ROBO1		y	y
SH3GLB1			
MANEAL		y	y
SMS			
NUCB2	y		
TFRC		y	y
NIPSNAP3A			
PTK7		y	y
UBXN1			
HNRNPR			
NT5E			
CHMP2A	y		
ACTR1A			
IRGQ			
TSG101			
AKR1A1	y		y
BUB3			
PPP2R1A			
UBA2			
DARS			
HNRNPK			
FNTA			
G3BP1			
PGM3			
SARS			
PSMD14	y		
PSMC3	y		
PRDX4	y		
PSMD5			
PSMD9			
EIF2S1			
MVP	y	y	
ANXA6	y	y	y
ZYX		y	y
C21orf33	y		
S100A16		y	y

Supplementary Figure. 3, related to Figure. 3 Generation of an LCSS for the ASCL1^{High} and NEUROD1^{High} NE-Lung cancer subtypes

a Transcriptomic clustering of genome wide microarray dataset in 39 NE-lung cancer cell lines[2] based on the AS/ND-LCSS gene expression markers. Pink dots indicate the ASCL1^{High} cells, and blue dots indicate the NEUROD1^{High} lines. **b** Transcriptomic clustering of genome wide microarray dataset in 23 SCLC patient sample cohort[3] based on the AS/ND-LCSS gene expression markers (the pink bar indicates the AS-LCSS genes that are clustered with ASCL1 whereas the blue bar indicates the ND-LCSS genes that are clustered with NEUROD1). **c** Summary table of the comprehensive transcriptome analysis. Y, the gene was clustered together with the corresponding transcription factor. N, not clustered with the corresponding transcription factor.

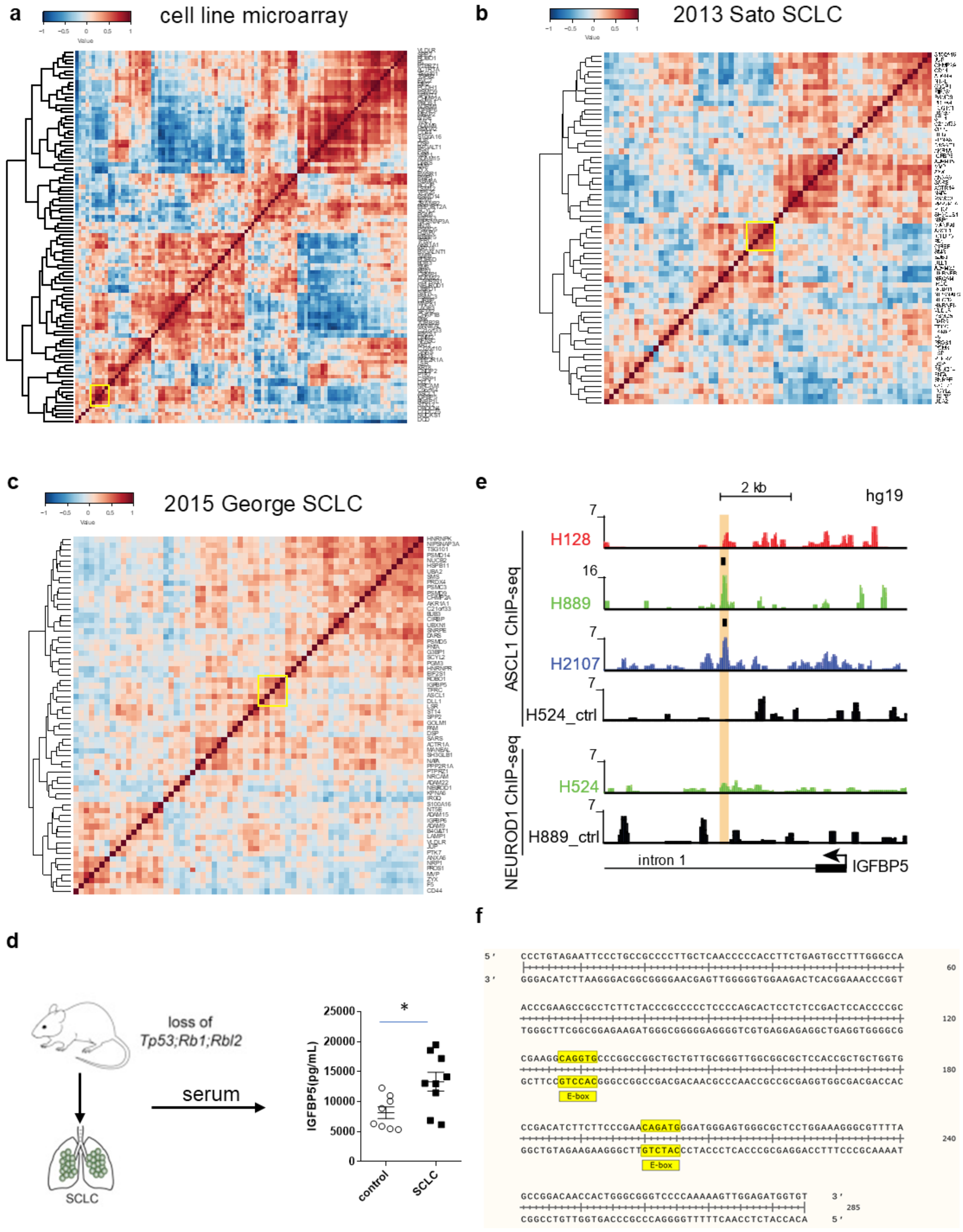

Supplementary Figure. 4, related to Figure. 4 Identification of IGFBP5 as a direct transcriptional target of ASCL1

a-c Correlation heatmap illustrating co-expression patterns of AS/ND-LCSS genes and ASCL1/NEUROD1. Data were extracted from three independent SCLC transcriptomic datasets as indicated. **d** ELISA analyses of IGFBP5 levels in the serum from the control and TCKO SCLC mice (n = 8-9). Unpaired two-tailed t-test. $^{*}P < 0.05$. e and f IGFBP5 is a transcriptional target of ASCL1. **e** Genome browser tracks from the human hg19 assembly showing the ASCL1 binding sites by ChIP-seq analyses in $ASCL1^{High}$ SCLC cells (H128, H889, and H2107) and a control $NEUROD1^{High}$ cell line (H524). The lower panel indicates the NEUROD1 binding sites in the genome of a $NEUROD1^{High}$ SCLC cell (H524) and a control $ASCL1^{High}$ cell line (H889). The P5 region (from +3.2 kb to +3.5 kb) is shown, which is located in the first intron of the *IGFBP5* gene (P5 genomic coordinates are 216692338-216692054 (GRCh38.p12 Primary Assembly). The black ticks above the tracks indicate that significant enrichment at these peaks was detected. ChIP-seq data (GEO: GSE69398) are from Borromeo et al., 2016. **f** DNA sequences of IGFBP5-P5-Luc where the E-box motifs are highlighted in yellow.

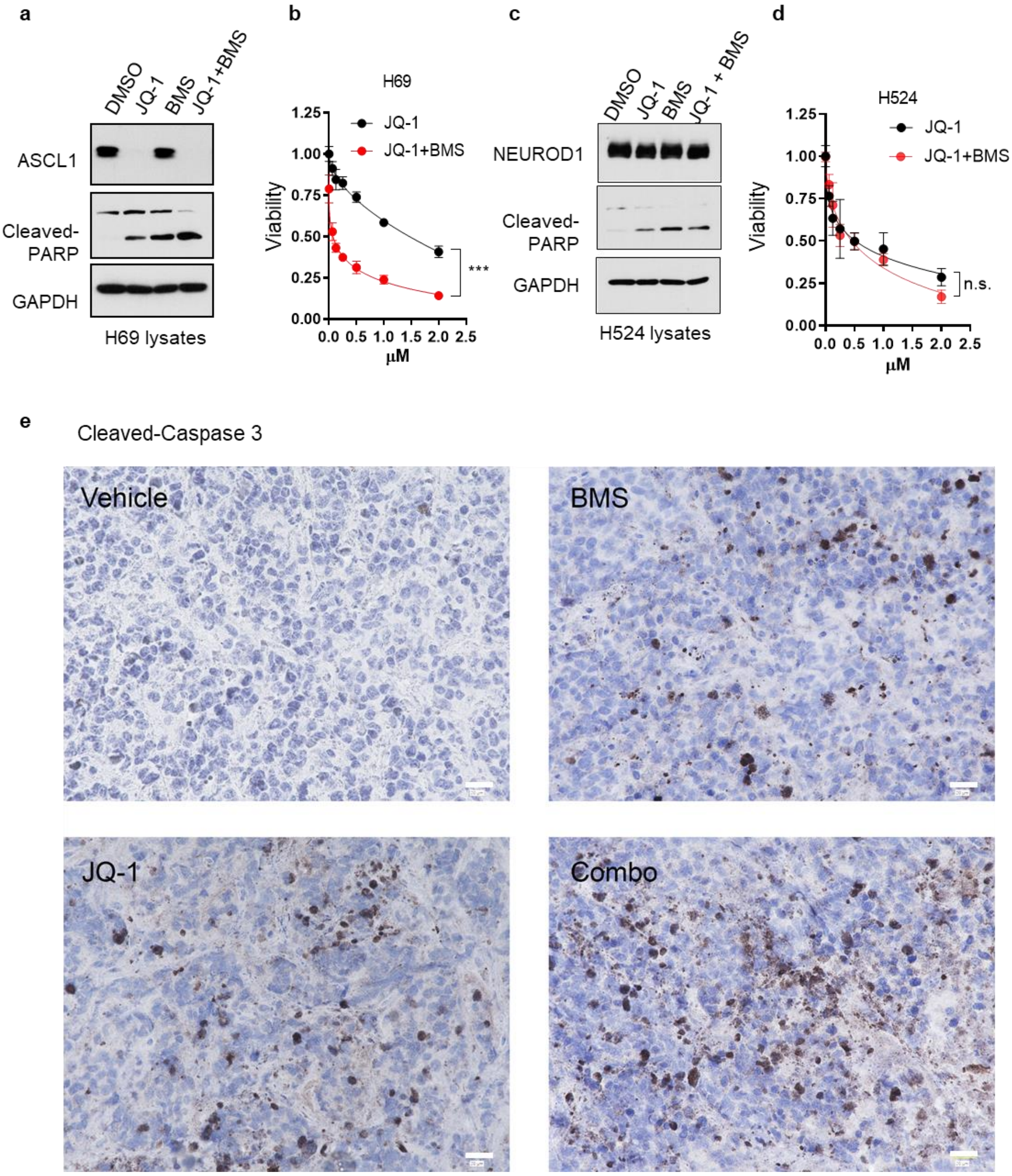

Supplementary Figure. 5, related to Figure. 5 JQ-1 treatment sensitizes ASCL1^{High} SCLC cells to IGF-1R inhibitors.

a, c Immunoblotting experiments of H69 cells (ASCL1^{High}) (**a**) and H524 cells (NEUROD1^{High}) (**c**) treated with DMSO, JQ-1 (1 μM), BMS-754807 (1 μM) and JQ-1 + BMS-754807 (1 μM each) for 48 hours. **b, d** Cell viability of H69 cells (n = 4) (**b**) and H524 cells (n = 4) (**d**) treated with JQ-1 or JQ-1+BMS-754807 at the indicated

concentrations for 72 hours. Unpaired two-tailed *t*-test. *** $P < 0.001$ **e** JQ-1+BMS-754807 combination treatment resulted in enhanced cell apoptosis in H2081 xenograft tumors. Representative images of Immunohistochemistry (IHC) (cleaved-caspase 3) analysis of H2081 xenograft tumor tissues. Bar 20 μm.

Fig. 4b

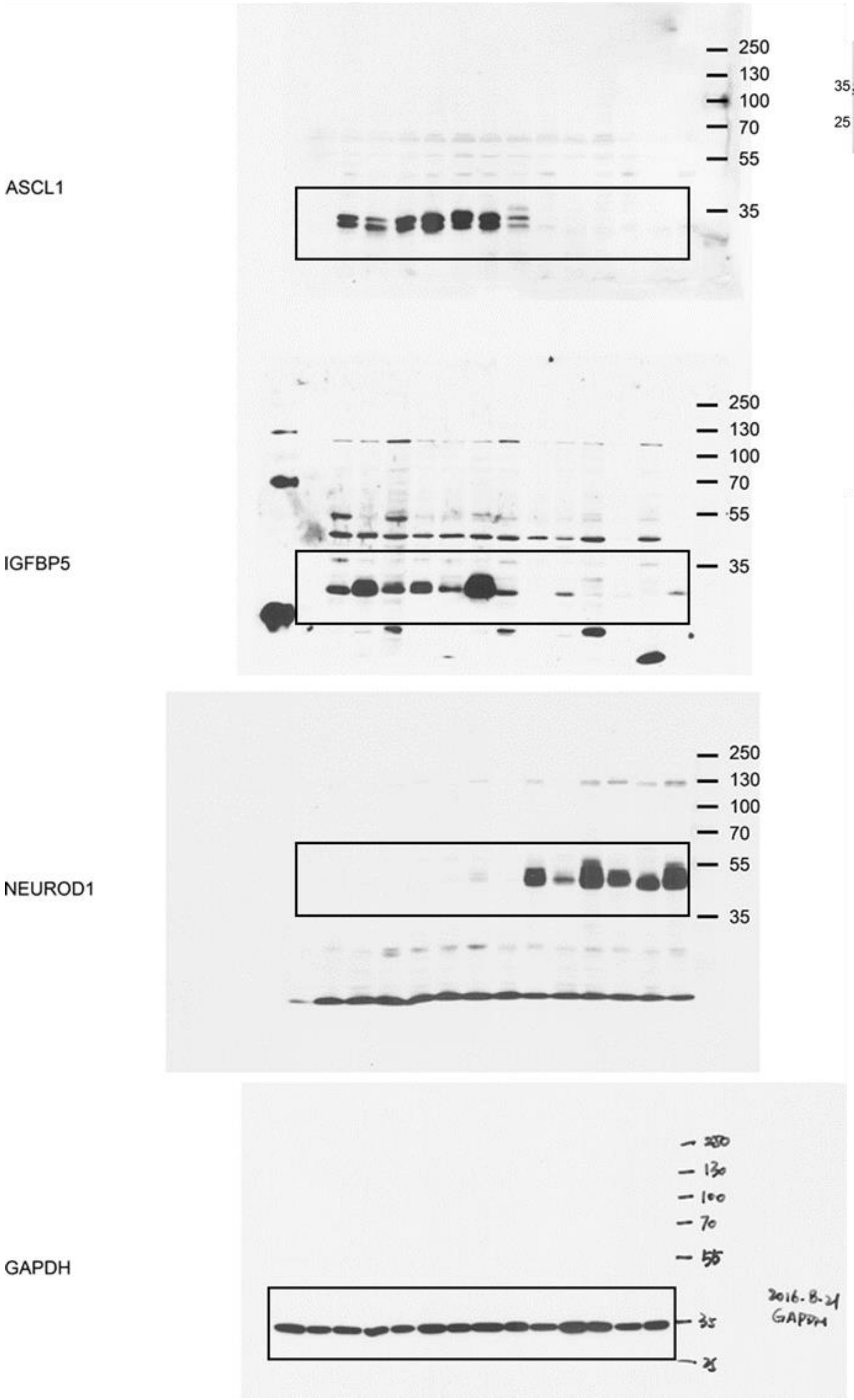

Fig. 4c

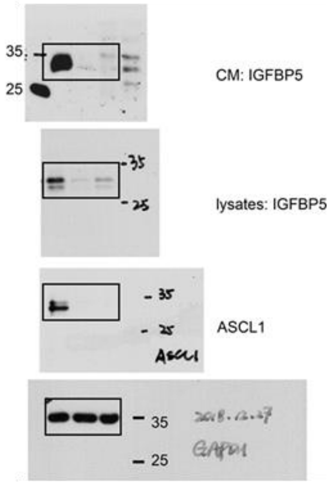

Fig. 4e

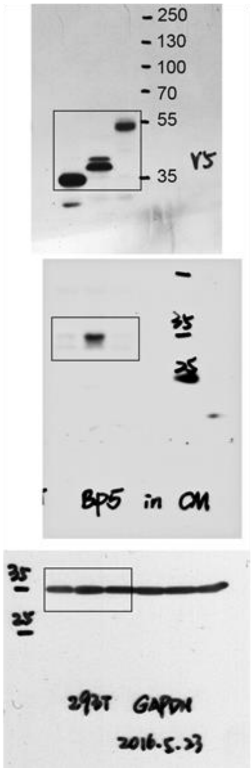

Supplementary Figure. 6. Fully uncropped versions of all gels and blots.

Fig. 5a

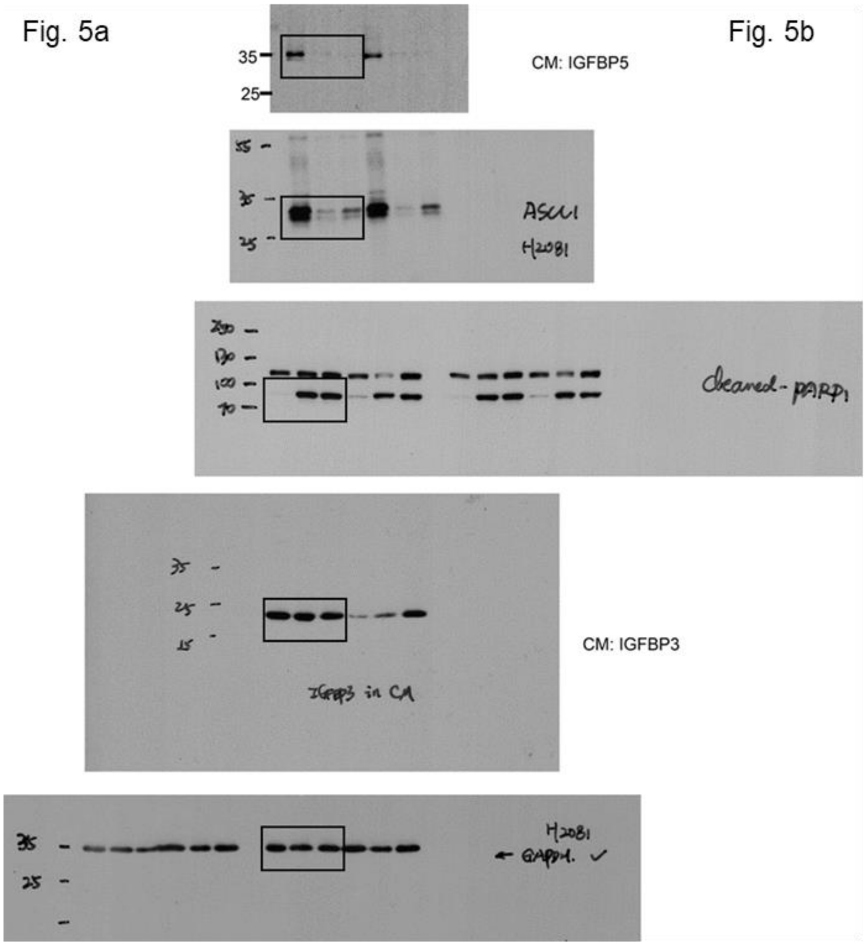

Fig. 5b

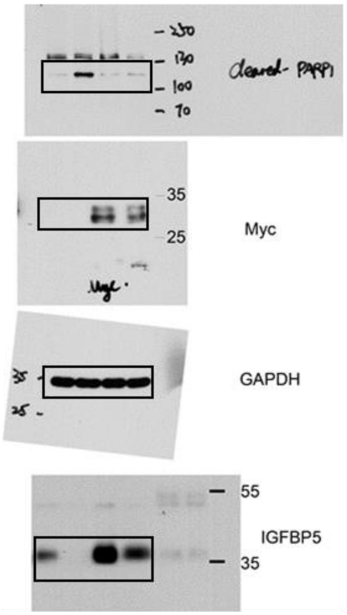

Fig. 5c

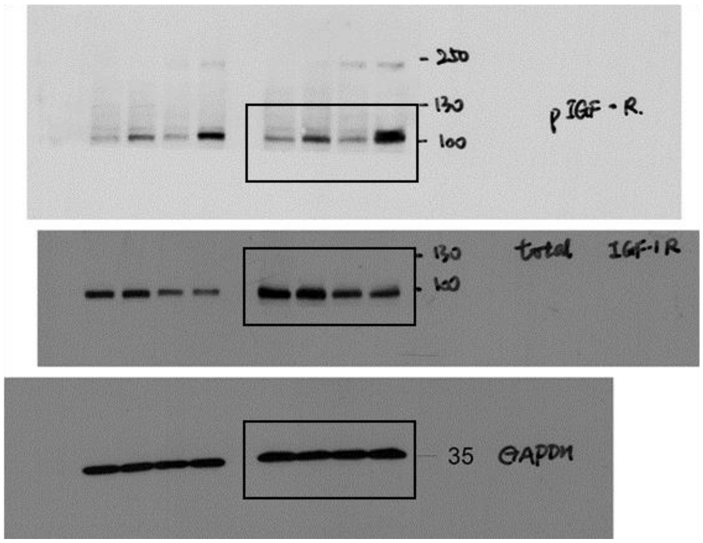

Fig. 5d

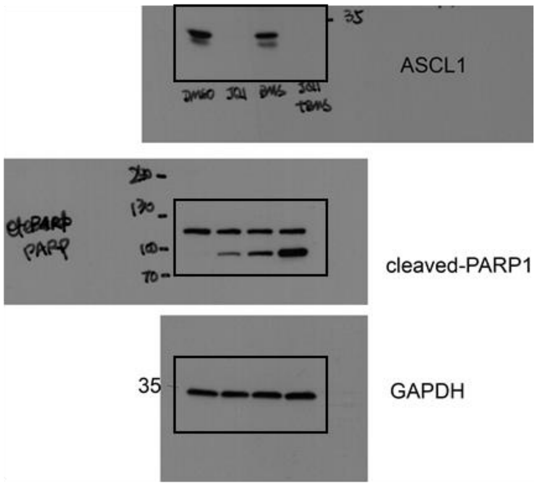

Supplementary Figure. 6 (continued). Fully uncropped versions of all gels and blots.

Supplementary Fig. 5a

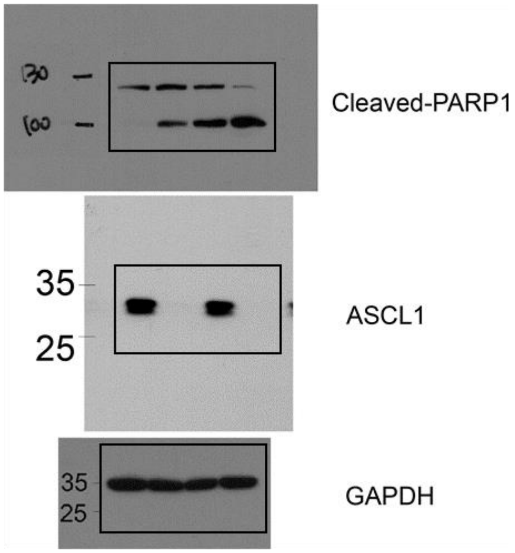

Supplementary Fig. 5c

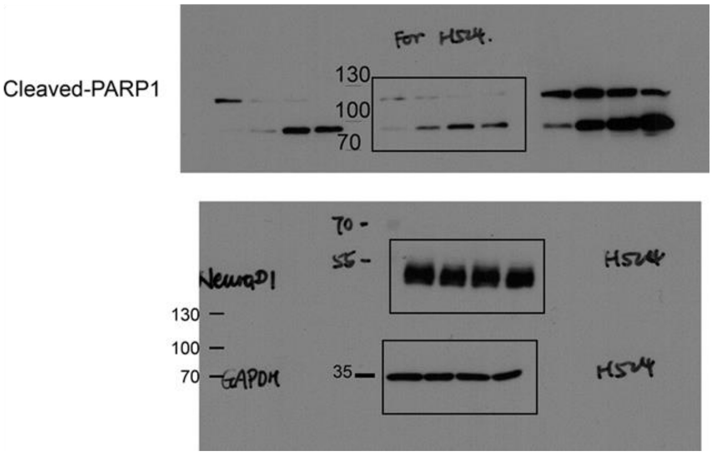

Supplementary Figure. 6 (continued). Fully uncropped versions of all gels and blots.

Supplementary Table. 1

The Pearson correlation coefficient for the correlation analysis between the secretome of NSCLC and NE-lung cancers.

	HBEC34-KT	HCC4018	H2081	HBEK3-KT	H1993	H2073
HBEC34-KT	1	0.311013	0.345129	0.988494	0.390833	0.398687
HCC4018	0.311013	1	0.749287	0.293786	0.58423	0.491554
H2081	0.345129	0.749287	1	0.32681	0.623733	0.504776
HBEK3-KT	0.988494	0.293786	0.32681	1	0.383709	0.402679
H1993	0.390833	0.58423	0.623733	0.383709	1	0.767335
H2073	0.398687	0.491554	0.504776	0.402679	0.767335	1

Supplementary Table. 2

DNA sequences for ASCL1 shRNAs and Lentiviral vectors.

**TRCN0000013550**	shASCL1#1
Region:CDS	Clone ID:NM_004316.1-1023s1c1
Sequence:CCGGCAACTACTCCAACGACTTGAACTCGAGTTCAAGTCGTTGGAGTAGTTGTTTTT	
**TRCN0000244309**	shASCL1#2
Region:CDS	Clone ID:NM_004316.3-955s21c1
Sequence:CCGGCAACCGCGTCAAGTTGGTCAACTCGAGTTGACCAACTTGACGCGGTTGTTTTTG	
**Lentiviral Gateway® destination vector for expression of a C-terminally V5-tagged protein.**	
pLenti6.3 V5-DEST	
Sequence: https://www.snapgene.com/local/fetch.php?set=gateway_cloning_vectors&plasmid=pLenti6.3_V5-DEST	
**Lentiviral plasmid (Δ8.9)**	
Sequence: https://www.addgene.org/browse/sequence_vdb/2221/	
**Lentiviral plasmid (VSVG)**	
Sequence: https://www.addgene.org/8454/sequences/	

Supplementary Table 3

Primers.

**Primers for Luciferase Constructs**		
	**Sequence (5'->3')**	
pGL4-P1-Luc	TATGGTACCTCTCCAGACTTTTAGGGGAGAAATTC	forward
	TATCTCGAGCCAGTTTGTAGCTGCAATTTGAGC	reverse
pGL4-P2-Luc	TATGGTACCATTGATTTGTTCCTACCTTACCAAGC	forward
	TATCTCGAGCCAGTTTGTAGCTGCAATTTGAGC	reverse
pGL4-P3-Luc	TATGGTACCGTTTTCACCCTTCTCCGGAC	forward
	TATCTCGAGTCCTTGACCAGCTCGCAGCCCA	reverse
pGL4-P4-Luc	TATGGTACCCTTCATCTTGGGGGATGTGGATTT	forward
	TATCTCGAGGGATCTTGCTTGGGACTGAAGTGT	reverse
pGL4-P5-Luc	TATGGTACCCCCTGTAGAATTCCCTGCCG	reverse
	TATCTCGAGACACCATCTCCAACTTTTTGGG	forward
pGL4-P6-Luc	TATGGTACCCTTTGGCAAACACTGCCAGAT	reverse
	TATCTCGAGTGAGACACATCTGCCTATGAAAG	forward
pGL4-P7-Luc	TATGGTACCTCTTTCAGCAGGAGAGGAGAGA	reverse
	TATCTCGAGTGTTTCAATTGGAAGTGCTGTTCT	forward
pGL4-P8-Luc	TATGGTACCTAGCCTTTGAAATCCGGTGT	reverse
	TATCTCGAGGGAAGGGGGAGCATGCTTAG	reverse
pGL4-P9-Luc	TATGGTACCTTCCTATGTGTACAGTTATCG	forward
	TATCTCGAGATCTGAGGCTCCTTGGACT	reverse
pGL4-P10-Luc	TATGGTACCTGCAGCCTCCAACTCCT	forward
	TATCTCGAGTCCCTTGCCAACCCTA	reverse
pGL4-P5-Luc-Del1	CCGCCGAAGGCCCGGCCGGC	forward
	GCCGGCCGGGCCTTCGGCGG	reverse

pGL4-P5-Luc-Del2	CGACATCTTCTTCCCGAAGGATGGGAGTGGGC	forward
	GCCCACTCCCATCCTTCGGGAAGAAGATGTCG	reverse
**Primers for real time PCR**		
RT-hIGFBP5	GTGCTGTGTACCTGCCCAAT	forward
	CGTCAACGTACTCCATGCCT	reverse
**Other Primers**		
pLenti-ASCL1-V5	GGGGACAAGTTTGTACAAAAAAGCAGGCTATGGAAAGCTCTGCCAAGAT	forward
pLenti-ASCL1-V5	GGGGACCACTTTGTACAAGAAAGCTGGGTCGAACCAGTTGGTGAAGTCGA	reverse
pLenti-NEUROD1-V5	GGGGACAAGTTTGTACAAAAAAGCAGGCTATGACCAAATCGTACAGCGA	forward
pLenti-NEUROD1-V5	GGGGACCACTTTGTACAAGAAAGCTGGGTCATCATGAAATATGGCATTGA	reverse

Supplementary References

1. Hu R, Huffman KE, Chu M, Zhang Y, Minna JD, Yu Y. Quantitative Secretomic Analysis Identifies Extracellular Protein Factors That Modulate the Metastatic Phenotype of Non-Small Cell Lung Cancer. *J Proteome Res* **15**, 477-486 (2016).

2. Augustyn A*, et al.* ASCL1 is a lineage oncogene providing therapeutic targets for high-grade neuroendocrine lung cancers. *Proc Natl Acad Sci U S A* **111**, 14788-14793 (2014).

3. Sato T*, et al.* PRC2 overexpression and PRC2-target gene repression relating to poorer prognosis in small cell lung cancer. *Scientific reports* **3**, 1911 (2013).
