# Supplementary material for: Subtype-specific secretomic characterization of pulmonary neuroendocrine tumor cells
Source: Nat Commun. 2019 Jul 19;10:3201. doi: 10.1038/s41467-019-11153-5 (PMC6642156; doi:10.1038/s41467-019-11153-5)
Supplement: Supplementary file 2 — Description of Additional Supplementary Files [file 41467_2019_11153_MOESM2_ESM.docx]

**Description of Supplementary Files**

**File Name:** **Supplementary Data 1.**

**Description:** Quantitative Secreted Protein Profiling of the conditioned medium from HBEC34-KT, CC4018 and H2081 cells. This file contains all quantitation data for the proteins identified, including protein accession number and description, normalised TMT 6-plex reporter ion intensity distribution for every protein in each replicate.

**File Name:** **Supplementary Data 2.**

**Description:** Quantitative Secreted Protein Profiling of the conditioned medium from HBEC34-KT, H889, H1092, H69, H2107, H128 cells (default~rq_126_sn sum - default~rq_131_sn sum). This file contains all quantitation data for the proteins identified, including protein accession number and description, normalised TMT 6-plex reporter ion intensity distribution for every protein in each cell line.

**File Name:** **Supplementary Data 3.**

**Description:** Quantitative Secreted Protein Profiling of the conditioned medium from HBEC34-KT, H378, H82, H2171, HCC970, H524 cells (default~rq_126_sn sum - default~rq_131_sn sum). This file contains all quantitation data for the proteins identified, including protein accession number and description, normalised TMT 6-plex reporter ion intensity distribution for every protein in each cell line.

**File Name:** **Supplementary Data 4.**

**Description:** Quantitative Secreted Protein Profiling of the 1,491 proteins commonly identified by secretome analyses in high-grade NE lung cancer and classic NSCLC.
