# Supplementary material for: Subtype-specific secretomic characterization of pulmonary neuroendocrine tumor cells
Source: Nat Commun. 2019 Jul 19;10:3201. doi: 10.1038/s41467-019-11153-5 (PMC6642156; doi:10.1038/s41467-019-11153-5)
Supplement: Supplementary file 7 — Reporting Summary [file 41467_2019_11153_MOESM7_ESM.pdf]

nature research

Corresponding author(s): Yonghao Yu

Last updated by author(s): May 21, 2019

Reporting Summary

Nature Research wishes to improve the reproducibility of the work that we publish. This form provides structure for consistency and transparency in reporting. For further information on Nature Research policies, see Authors & Referees and the Editorial Policy Checklist.

Statistics

For all statistical analyses, confirm that the following items are present in the figure legend, table legend, main text, or Methods section.

n/a	Confirmed	
☐	☒	The exact sample size (*n*) for each experimental group/condition, given as a discrete number and unit of measurement
☐	☒	A statement on whether measurements were taken from distinct samples or whether the same sample was measured repeatedly
☐	☒	The statistical test(s) used AND whether they are one- or two-sided<br>*Only common tests should be described solely by name; describe more complex techniques in the Methods section.*
☐	☒	A description of all covariates tested
☐	☒	A description of any assumptions or corrections, such as tests of normality and adjustment for multiple comparisons
☐	☒	A full description of the statistical parameters including central tendency (e.g. means) or other basic estimates (e.g. regression coefficient) AND variation (e.g. standard deviation) or associated estimates of uncertainty (e.g. confidence intervals)
☐	☒	For null hypothesis testing, the test statistic (e.g. *F*, *t*, *r*) with confidence intervals, effect sizes, degrees of freedom and *P* value noted<br>*Give P values as exact values whenever suitable.*
☒	☐	For Bayesian analysis, information on the choice of priors and Markov chain Monte Carlo settings
☒	☐	For hierarchical and complex designs, identification of the appropriate level for tests and full reporting of outcomes
☐	☒	Estimates of effect sizes (e.g. Cohen's *d*, Pearson's *r*), indicating how they were calculated

Our web collection on statistics for biologists contains articles on many of the points above.

Software and code

Policy information about availability of computer code

Data collection	We have specified all open source or commercial software to collect data in the Method section.
Data analysis	We have specified all open source or commercial software to collect data in the Method section.

For manuscripts utilizing custom algorithms or software that are central to the research but not yet described in published literature, software must be made available to editors/reviewers. We strongly encourage code deposition in a community repository (e.g. GitHub). See the Nature Research guidelines for submitting code & software for further information.

Data

Policy information about availability of data

All manuscripts must include a data availability statement. This statement should provide the following information, where applicable:

- Accession codes, unique identifiers, or web links for publicly available datasets
- A list of figures that have associated raw data
- A description of any restrictions on data availability

The mass spectrometry data have been deposited to the ProteomeXchange Consortium (https://www.ebi.ac.uk/pride/archive/) via the PRIDE partner repository with the dataset identifiers: PXD013298 (Username: reviewer45259@ebi.ac.uk; Password: KvX08veo) and PXD013267 (Username: reviewer69919@ebi.ac.uk; Password: LyVrqLw9). Microarray, RNA-seq and ChIP-seq data for human patient samples and cell lines were obtained from published literatures5, 10, 16, 33. The gene expression microarray data from the lung cancer cell lines were downloaded from the Gene Expression Omnibus (https://www.ncbi.nlm.nih.gov/geo/) with accession number GSE32036[https://www.ncbi.nlm.nih.gov/geo/query/acc.cgi?acc=GSE32036]. The gene expression microarray data from the human SCLC tumor tissue was downloaded from GEO with accession numbers GSE43346[https://www.ncbi.nlm.nih.gov/geo/query/acc.cgi?acc=GSE43346]. ChIP-seq libraries were sequenced on an Illumina High-Seq 2000 or Illumina GAIIx (GSE69398[https://www.ncbi.nlm.nih.gov/geo/query/acc.cgi?acc=GSE69398]). Computer code and all the other data supporting the findings of this study are available within the article and its supplementary information files and from the corresponding author upon request. The source data underlying Figs 2b, 3a, 3d-i, 4d, 4g, 4h, 5e, 5f, 5g-i and Supplementary Figs 1d, 1f, 2c, 4d, 5b and 5d are provided as a Source Data file. Fully uncropped versions of all gels and blots are shown in Supplementary Figure 6.

Field-specific reporting

Please select the one below that is the best fit for your research. If you are not sure, read the appropriate sections before making your selection.

☒ Life sciences ☐ Behavioural & social sciences ☐ Ecological, evolutionary & environmental sciences

For a reference copy of the document with all sections, see nature.com/documents/nr-reporting-summary-flat.pdf

Life sciences study design

All studies must disclose on these points even when the disclosure is negative.

Sample size	Sample size was based on our previous experience in the experimental approach, and also taking into account feasibility, in obtaining reliable results.
Data exclusions	No samples or animals were excluded from the analysis.
Replication	Findings were reliably reproduced.
Randomization	Mice implanted with xenograft tumors were randomized into experimental groups.
Blinding	Immunoblotting assays were exposed with the same settings without the investigatores knowing the order of the samples. They were identified later with markers assigned to them.

Reporting for specific materials, systems and methods

We require information from authors about some types of materials, experimental systems and methods used in many studies. Here, indicate whether each material, system or method listed is relevant to your study. If you are not sure if a list item applies to your research, read the appropriate section before selecting a response.

Materials & experimental systems

n/a	Involved in the study	
☐	☒	Antibodies
☐	☒	Eukaryotic cell lines
☒	☐	Palaeontology
☐	☒	Animals and other organisms
☒	☐	Human research participants
☒	☐	Clinical data

Methods

n/a	Involved in the study	
☒	☐	ChIP-seq
☒	☐	Flow cytometry
☒	☐	MRI-based neuroimaging

Antibodies

Antibodies used	IIGF-1R (#3027, CST, dilution 1:1000), p-IGF-1R (Y1135/1136) (#3024, CST, dilution 1:1000), IGFBP5 (p-19, Santa Cruz, dilution 1:750), V5 (A190-120A, Bethyl Laboratories, Inc., dilution 1:2000), Myc-Tag (9B11) (#2276, CST, dilution 1:2000), GAPDH (sc-32233, Santa Cruz, dilution 1:5000), NEUROD1 (ab60704, Abcam, dilution 1:1000), IGFBP3 (09-180, EMD Millipore, dilution 1:1000), Cleaved Caspase-3 (Asp175) (#9661s, CST, dilution 1:200), Cleaved PARP (Asp214) (#9546s, CST, dilution 1:1000) were obtained from commercial sources. ASCL1 (J.E.J. lab TX518, dilution 1:5000) antibody was generated as described.
Validation	For antibodies that have been validated in our previous studies, see Ding M, Bruick RK, Yu Y. Secreted IGFBP5 mediates mTORC1-dependent feedback inhibition of IGF-1 signalling. Nature cell biology 18, 319-327 (2016); Borromeo MD, et al. ASCL1 and NEUROD1 Reveal Heterogeneity in Pulmonary Neuroendocrine Tumors and Regulate Distinct Genetic Programs. Cell reports 16, 1259-1272 (2016). For antibodies obtained from commercial sources listed in the above section, validation was performed by the vendors.

Eukaryotic cell lines

Policy information about cell lines

Cell line source(s)	All lung cell lines used in this study were obtained from the Hamon Cancer Center Collection (University of Texas Southwestern Medical Center, Dallas, TX).
Authentication	All cell lines have been DNA fingerprinted by DNA Genotyping Core (University of Texas Southwestern Medical Center, Dallas, TX) using The GenePrint® 10 PowerPlex 1.2 kit System (Promega).

Mycoplasma contamination	All cell lines are found to be mycoplasma free using the e-Myco kit (Boca Scientific).
Commonly misidentified lines (See ICLAC register)	No commonly misidentified cell lines were used.

Animals and other organisms

Policy information about studies involving animals; ARRIVE guidelines recommended for reporting animal research

Laboratory animals	Xenograft animal species: NOD/SCID; sex: male; age: 4-6 weeks old. The SCLC mouse model with conditional deletions in Trp53 (p53), Rb1, and Rbl2 (p130) was described previously (Borromeo MD, et al. ASCL1 and NEUROD1 Reveal Heterogeneity in Pulmonary Neuroendocrine Tumors and Regulate Distinct Genetic Programs. Cell reports 16, 1259-1272 (2016)).
Wild animals	This study did not involve wild animal.
Field-collected samples	The study did not involve samples collected from the field.
Ethics oversight	All animal procedures were reviewed and approved by the institutional animal care and use committee at UT Southwestern medical center.

Note that full information on the approval of the study protocol must also be provided in the manuscript.
